# Mechanism of external K^+^ sensitivity of KCNQ1 channels

**DOI:** 10.1085/jgp.202213205

**Published:** 2023-02-21

**Authors:** Astghik Abrahamyan, Jodene Eldstrom, Harutyun Sahakyan, Nare Karagulyan, Liana Mkrtchyan, Tatev Karapetyan, Ernest Sargsyan, Matthias Kneussel, Karen Nazaryan, Jürgen R. Schwarz, David Fedida, Vitya Vardanyan

**Affiliations:** 1 Molecular Neuroscience Group, Institute of Molecular Biology, National Academy of Sciences of the Republic of Armenia, Yerevan, Armenia; 2 Department of Anesthesiology, Pharmacology and Therapeutics, University of British Columbia, Vancouver, BC, Canada; 3 Laboratory of Computational Modeling of Biological Processes, Institute of Molecular Biology of National Academy of Sciences of the Republic of Armenia, Yerevan, Armenia; 4 Institute for Molecular Neurogenetics, Center for Molecular Neurobiology Hamburg, Hamburg, Germany

## Abstract

KCNQ1 voltage-gated K^+^ channels are involved in a wide variety of fundamental physiological processes and exhibit the unique feature of being markedly inhibited by external K^+^. Despite the potential role of this regulatory mechanism in distinct physiological and pathological processes, its exact underpinnings are not well understood. In this study, using extensive mutagenesis, molecular dynamics simulations, and single-channel recordings, we delineate the molecular mechanism of KCNQ1 modulation by external K^+^. First, we demonstrate the involvement of the selectivity filter in the external K^+^ sensitivity of the channel. Then, we show that external K^+^ binds to the vacant outermost ion coordination site of the selectivity filter inducing a diminution in the unitary conductance of the channel. The larger reduction in the unitary conductance compared to whole-cell currents suggests an additional modulatory effect of external K^+^ on the channel. Further, we show that the external K^+^ sensitivity of the heteromeric KCNQ1/KCNE complexes depends on the type of associated KCNE subunits.

## Teaser

Occupation of the uppermost ion-binding site of the selectivity filter of K^+^ channels by external K^+^ promotes channel activity. Such a phenomenon is not observed in KCNQ1 voltage-gated channels, allowing a deeper understanding of their conduction and permeability mechanism.

## Introduction

KCNQ1 K^+^ channels are expressed in several human excitable and epithelial tissues (Chouabe et al., 1997) where they play a key role in the regulation of cellular excitability and transepithelial ion transport (Jespersen et al., 2005; Abbott, 2014). Many mutations of the KCNQ1 gene are associated with diseases of the human heart (Wang et al., 1996; Duggal et al., 1998; Peroz et al., 2008; Hedley et al., 2009), inner ear (Chouabe et al., 1997), endocrine system (Torekov et al., 2014; Tommiska et al., 2017), and brain (Goldman et al., 2009). Despite the enormous therapeutic significance of these channels, our understanding of their subunit composition, biophysical and pharmacological features, as well as the mechanisms of their regulation remains incomplete.

KCNQ1 channels are multisubunit complexes composed of pore-forming α subunits arranged in a symmetrical fourfold structure in characteristic domain-swapped configuration (Sun and MacKinnon, 2020) and KCNE1–5 β subunits. The cellular Ca^2+^-sensor calmodulin is an integral constituent of native and heterologously expressed KCNQ1 channels (Ghosh et al., 2006; Sachyani et al., 2014; Sun and MacKinnon, 2020). KCNE proteins are thought to dock between voltage-sensor domains and the pore domain of α subunits (Sun and MacKinnon, 2020) whereas calmodulin molecules interact with the channel from the cytoplasmatic side of the cell membrane (Shamgar et al., 2006). Calmodulin is strictly required for proper channel assembly and trafficking (Ghosh et al., 2006; Shamgar et al., 2006). Phosphatidylinositol-4,5-bisphosphate (PIP2)-signaling lipid binds to KCNQ1 complexes as well (Sun and MacKinnon, 2020) and plays an essential role in channel gating (Loussouarn et al., 2003). The biophysical and pharmacology properties of KCNQ1 complexes vary quite considerably depending on the type (Barhanin et al., 1996; Jespersen et al., 2005) of associated KCNE protein and likely on their quantity as recent studies on KCNE1 subunit indicate (Wang et al., 2020). Intracellular second messengers such as free Ca^2+^ ions (Tohse, 1990; Shamgar et al., 2006) and adenosine nucleotides (Li et al., 2013; Kienitz and Vladimirova, 2015), as well as membranes lipids (Loussouarn et al., 2003; Zaydman et al., 2013; Liin et al., 2015b) and protein kinase A (Yazawa and Kameyama, 1990) exert a pronounced modulatory action on these channels.

A substantial inhibitory effect of external K^+^ (K^+^_o_) on KCNQ1 is a particularly intriguing feature of these channels (Larsen et al., 2011), reported for the first time nearly two decades ago (Yang and Sigworth, 1998). Such regulation may have a central role in the modulation of the endocochlear potential and K^+^ recycling in the inner ear, given the strong expression of these channels in strial marginal cells (MC; Knipper et al., 2006; Wang et al., 2015) and dark cells of the vestibular organ (Marcus and Shen, 1994). K^+^_o_ dependency on the KCNQ1 channels can have a significant impact on the physiology of gastrointestinal organs where these channels are abundantly expressed (Chouabe et al., 1997; Liin et al., 2015a). For instance, KCNQ1/KCNE3 heteromeric channels are localized in the basolateral membranes of epithelial cells of the small intestine (Preston et al., 2010). The concentration of the luminal K^+^ in the small intestine increases several hours after consumption of food, which triggers passive absorption of K^+^ into the bloodstream through a paracellular pathway (Agarwal et al., 1994). During this process, the epithelial cells will be exposed to elevated [K^+^]_o_. Given the well-recognized stabilizing influence of external K^+^_o_ on the conductive properties of most K^+^ channels, understanding the molecular mechanism of the inhibitory action of K^+^_o_ on KCNQ1 has the potential to provide new insights into basic principles of K^+^ channel permeability and gating.

The exact molecular mechanism of how K^+^_o_ modulates KCMQ1 channels is currently unclear. It has been proposed that the augmentation of channel’s fast inactivation underlies the inhibitory action of K^+^_o_ (Larsen et al., 2011). However, a significant K^+^_o_-dependent inhibition of the non-inactivating KCNQ1/KCNE1 complex expressed in *Xenopus laevis* oocytes (Yang and Sigworth, 1998) and CHO cells (Wang et al., 2015) has also been reported. The negative charge of the E290 residue was proposed as an essential element of the K^+^_o_ sensitivity (Wang et al., 2015), but the corresponding mechanism is not clear.

Here, we have explored the K^+^_o_ dependency of KCNQ1 channels in more detail and provide a molecular basis underlying this phenomenon. We first show that the fast inactivation of the channel, as well as the negatively charged residues in the turret region of the pore, plays no principal role. An extensive mutational analysis and subsequent molecular dynamics (MD) simulations revealed the involvement of the selectivity filter (SF) in this process. MD simulations also demonstrate that the enhanced occupancy of the uppermost K^+^ binding site (S_0_) of the SF operating in a specific conducting mode triggers the modulation of the channel conductance. In accordance with these findings, a marked reduction in unitary conductance at elevated K^+^_o_ conditions was observed in single-channel recordings. Furthermore, we show that K^+^_o_ sensitivity of distinct KCNQ1 complexes varies considerably depending on the type of associated KCNE subunit.

## Materials and methods

### Constructs and mutagenesis

Mutations in the human KCNQ1 gene, cloned into oocyte expression vector pGEM-He-Juell, were introduced by overlap PCR method as well as using the QuickChange kit (Stratagene). Each mutation was verified by DNA sequencing. mRNA synthesis was made with the T7 mMessage mMachine transcription kit (Ambion). mRNA was then analyzed in gel electrophoresis and quantified by RNA 6000 Nano kit (Agilent Technologies) or spectroscopic method. The EQQ construct was generated as previously described (Murray et al., 2016). It was subcloned into pGem He-Juell vector using HindIII and XbaI restriction sites. All new constructs were confirmed by sequencing.

### Channel expression

*Xenopus* frogs (Nasco) were kept in the animal facility of the Institute of Molecular Biology National Academy of Sciences of the Republic of Armenia according to the guidelines of the local animal welfare authorities. Oocytes were removed surgically and defolliculated with 2–3 mg/ml collagenase (Roche) in Ca^2+^-free OR2 solution containing 82.5 mM NaCl, 2 mM KCl, 1 mM MgCl_2_, and 5 mM HEPES, pH 7.5. Stage IV or V oocytes were selected and injected with 2–50 ng cRNA on the next day. Injections were performed using an oocyte manual microinjection pipette (Drumond Scientific Company). Mixtures of KCNQ1/KCNE1, KCNQ1/KCNE2, and KCNQ1/KCNE3 cRNAs with different molar ratios of α to β were made prior to injection. Electrophysiological experiments were performed 2–7 d after injection. Oocytes were incubated in OR2 solution supplemented with 2 mM CaCl_2_, 5 mM sodium pyruvate, and 50 mg/ml gentamycin at 17°C conditions prior to electrophysiological recording.

For single-channel recordings, *ltk*-mouse fibroblast cells (LM) were cultured and plated for experiments as previously described (Murray et al., 2016). Cells were transfected using Lipofectamine2000 (Thermo Fisher Scientific) as per the manufacturer’s protocol. The EQQ constructs were transfected with GFP in a 2:0.7 ratio (in micrograms). F339A was GFP tagged and transfected alone at 1.5 μg/dish of cells.

### Electrophysiological recordings

TEVC recordings were performed at room temperature using Tec-03X or Turbo TEC-05 amplifiers (NPI Electronics) linked to Patchmaster software (HEKA Electronics) using a Instrutec digitizer for data acquisition and monitoring. Some experiments were conducted in a setup based on Axon 500B amplifier (Molecular Devices) linked to Patchmaster software (HEKA Electronics) through ITC-16 Computer Interface (Port). Borosilicate glass capillaries (WPI) were pulled to fabricate pipettes in PB/10 puller (Narishige). Tips of the recording electrodes were prefilled with 1% agar-3M KCl solution and backfilled with 3M KCl to prevent KCl leakage into the oocytes. Pipette resistances were 0.1–0.3 MΩ. Leakage current subtraction was not used due to the voltage-independent gating component in KCNQ1 channels. Recordings were performed in ND96 solutions containing (in mM) 96 NaCl, 2 KCl, 1.8 CaCl_2_, 1 MgCl_2_, and 5 HEPES, pH 7.5. Different K^+^-containing solutions were obtained via substitution of NaCl with KCl. 0.2 mM K^+^ solution contained (in mM) 0.2 KCl, 95.8 NaCl, 1.8 CaCl_2_, 1 MgCl_2_, and 5 HEPES, pH 7.5; 100 mM Na^+^ solution (nominally K^+^ free) contained 100 NaCl, 1.8 CaCl_2_, 1 MgCl_2_, and 5 HEPES, pH 7.5. In the latter solution, 100 mM Na^+^ was substituted by 100 mM K^+^, Rb^+^, or NMDG^+^ to get the corresponding solutions. All chemicals were from Sigma unless otherwise stated. Oocytes were recorded in a chamber with a volume of 0.35 ml. A five-channel perfusion system was used to exchange the extracellular solution. Oocyte perfusion at 2.5 ml/min flow rate was kept constant in all experiments. Measurements of the oocytes for determination of the concentration dependency of KCNQ1 channels were performed according to the following procedure. Oocytes expressing wild type or mutant channels were placed from the incubation solution (OR2 supplemented with Ca^2+^ [2 mM], Na-pyruvate [5 mM], and gentamicin [50 mg/ml]) into the recording chamber perfused with 0.2 mM K^+^ containing ND96 solution. After clamping the oocytes at holding potential (usually −100 mV), continuous depolarization pulses with +60 mV amplitude and 120 s interpulse intervals were applied. KCNQ1 current amplitudes in this solution usually stabilized after four to six consequent pulses, after which the values of the next three points were taken for 0.2 mM K^+^_o_. Then, the perfusion was switched to 2 mM K^+^ containing solution and the next three points were recorded. The experiments were continued according to this scheme until the last three points corresponding to 100 mM K^+^_o_ were recorded. Recordings that, for some reason, did not reach 100 mM K^+^ perfusion step were not analyzed. To estimate the unspecific currents, the water-injected oocytes from the parallel batch were recorded in an identical way. Recordings of these oocytes were done on the same day using the same solutions and by application of identical protocols and solution exchange schemes. Then, we averaged the unspecific currents values corresponding to three to four water-injected oocytes taken at specific cursor positions, which were identical to that of the analysis of wild type and mutant channels. Subsequently, these unspecific current values were subtracted from three data points for each K^+^_o_ concentration, and the data was plotted against time. The concentration–effect relationships were determined via averaging three points of the given concentration and correcting them for theoretical values, if not otherwise specified. Data points were then fitted to the Hill equation using Igor Pro software (WaveMetrics).

Single-channel recordings were acquired as previously published (Werry et al., 2013). Acquisitions were made using an Axopatch 200B amplifier, Digidata 1440A, and pClamp 10 software (Molecular Devices). Records were low-pass filtered at 2 kHz at acquisition using a 3 dB, four-pole Bessel filter, sampled at 10 kHz, and digitally filtered at 200 Hz before analysis. For single-channel recordings, the bath solution contained (in mM) 135 KCl, 1 MgCl_2_, 0.1 CaCl_2_, 10 dextrose, and 10 HEPES (pH 7.4 with KOH). The pipette solution contained (in mM) 6 NaCl, 129 MES, 1 MgCl_2_, 5 KCl, 1 CaCl_2_, and 10 HEPES (pH 7.4 with NaOH) or 97 KCl, 41.3 MES, 1 MgCl_2_, 1.9 NaCl, and 10 HEPES. Electrodes were pulled from thick-walled borosilicate glass (Sutter Instrument) using the linear multistage electrode puller (Sutter Instruments). Electrodes were coated with Sylgard (Dow Corning). After fire polishing, single-channel electrode resistances were between 40 and 60 MΩ. The *n* values refer to the number of individual cells recorded via TEVC or patch clamp methods.

### Computational electrophysiology simulations

CompEL (computational electrophysiology) method (Kutzner et al., 2011) with dual membrane configuration implemented in GROMACS was used to investigate ion permeations through the pore region of the KCNQ1 channel. Permeation of ions was driven by a transmembrane voltage, which was generated by charge imbalance (2 Cl ions) between compartments. The open-state cryo-EM structure of KCNQ1 (PDB ID: 6V01; Sun and MacKinnon, 2020) with removed KCNE3 was used in simulations. To keep the intracellular gate in the open state, terminal amino acids of the S5 and S6 helices were restrained. The protein membrane system was assembled via CHARMM-GUI and embedded into a POPC lipid bilayer surrounded by water and K^+^/Na^+^/Cl^−^ ions. Systems were minimized and equilibrated gradually releasing position restraints ending with 10 ns of NTP ensemble equilibrations. *N* values in the text refer to the number of separate MD simulations.

### Data analysis

**Figure S1.**
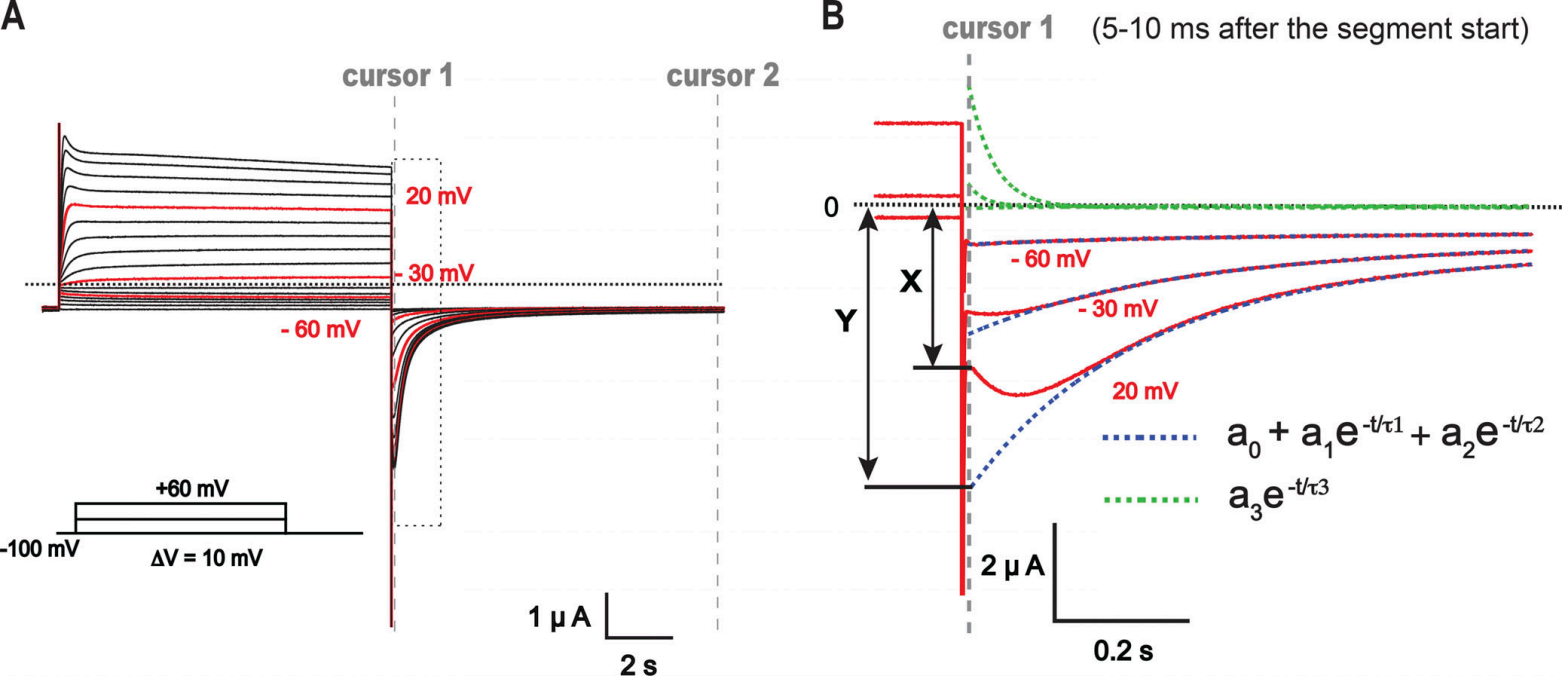
**The method of the estimation of fractional fast inactivation in KCNQ1 channels. (A)** Sample current traces in 20 mM external K^+^ conditions elicited by the voltage protocol shown below the traces. Calculation of the fractional inactivation for the red traces is demonstrated on the right side. The tail currents were fitted to three exponential functions of the form ao + a1e-t/τ1 + a2e-t/τ2 +a3e-t/τ3 between two cursors positioned immediately after capacitive transient (typically 5–10 ms after the segment start) and at 97% of the segment. **(B)** Enlarged view of the tail currents and corresponding exponential components of the fit. The sum of two exponential components with negative amplitudes (a1 and a2) together with constant amplitude a0 (black dashed curve) was extrapolated to the beginning of the segment. The intercept with the y axis was nominated as Y (represents total current amplitude). The initial current immediately after the capacitive transient was designated as X (represents the current of open channels at this time point). Fractional inactivation was calculated as 1 − X/Y.

Data were analyzed using Fitmaster (HEKA Electronics), Kaleidograph (Synergy Software), and Igor pro (Wavemetrics) software. Inactivation of wild type and mutant KCNQ1 channels was determined by fitting tail currents with two or three exponential functions with subsequent extrapolation of the fit to the beginning of the segment. Calculation of ratios was then performed as described in Fig. S1, adopted from early publication (Tristani-Firouzi and Sanguinetti, 1998). Voltage-dependent activation of T312C was determined from tail currents after their correction for inactivation. Data were then fitted to a Boltzmann function with offset as previously described (Ma et al., 2011). Concentration–response data were determined as follows: peak currents were determined from the entire depolarizing segment (+60 mV) for each external K^+^ concentration. Theoretical values were then subtracted assuming K^+^_in_ = 108 mM (Weber, 1999). Theoretical reduction of the current amplitude and absolute single-channel permeation P_K_ was calculated using Goldman–Hodgkin–Katz (GHK) flux equation of the form Ik = P_K_(VmF2/RT)[Ki − Koexp(−VmF/RT)/(1 − exp(−VmF/RT)], where P_K_ is the absolute single-channel permeability; Vm is the membrane potential; Ki and Ko are K^+^ concentrations in inside and outside solutions, respectively; Vm is the membrane voltage; and F, R, and T (293 K) have their usual meanings. Assuming that channels are not modified by extracellular K^+^, the ratio of current reduction in two external K^+^ concentrations K^+^_o_(C1), K^+^_o_(C2), is given by the equation: Ic1/Ic2 = [K^+^_in_(C1) − K^+^_o_(C1)exp(−VmF/RT]/[K^+^_in_(C2) − K^+^_o_(C2)exp(−VmF/RT)], where K^+^_o_(C1), K^+^_o_(C2), K^+^_in_(C1), and K^+^_in_(C2) are the outside and inside K^+^ concentrations, accordingly (Larsen et al., 2011). Data corrected for theoretical values were then fitted with a Hill function of the form I(C) = (1 − *Imax*) + (*Imax*/(1 + [C/IC_50_]^nH^), where *Imax* is the maximal inhibition, IC_50_ is the concentration of half-maximal inhibition, nH is the Hill coefficient, and C is the concentration. Permeability ratios were calculated as P_X_/P_K_ = exp(ΔErvZF/RT), where ΔErv is the mean difference of reversal potentials before and after exchange of extracellular cation, and Z, F, R, and T have their usual meanings. Exact P values are provided in Table S2.

To determine the conductance of EQQ, all-point histograms were generated from records containing the activity of a single channel at various voltages. Gaussian fits of the all-points histograms were made using Clampfit and the largest peak at each voltage was used to generate the conductance curves. Linear regression fitting and statistical analysis of the conductance curves were carried out using Prism 8 and their version of the analysis of covariance (ANCOVA; GraphPad Software).

### Online supplemental material

**Figure S2:**
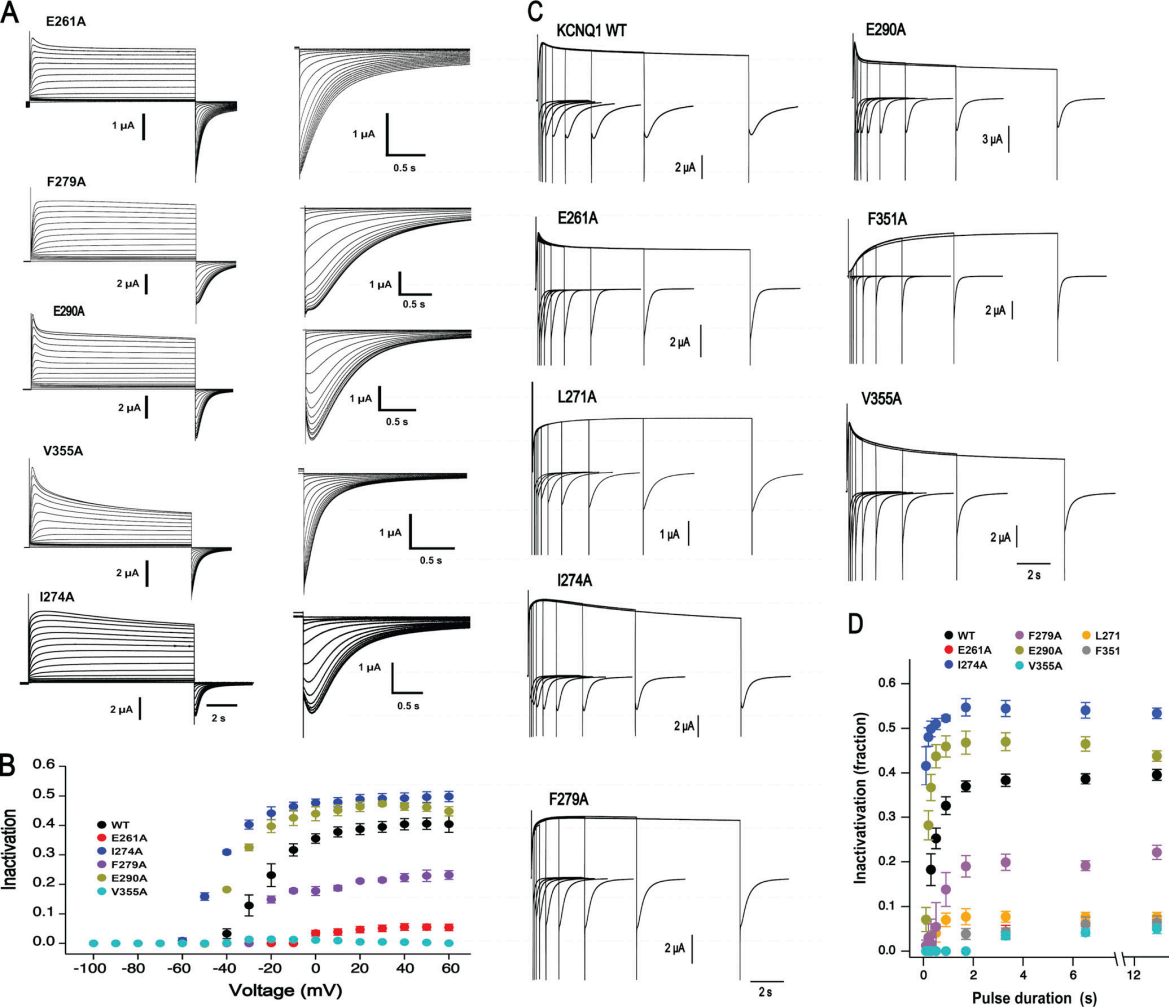
**The extent of fast inactivation depends on the duration and amplitude of applied depolarizing pulse.** **(A)** Sample current traces of mutants in response to depolarization pulse from holding potential −100 mV to +60 mV with 10 mV increments (left). Enlarged tail currents visualize hooks on the tail (right). **(B)** Analysis of fractional inactivation depending on activation potential. Tail currents were fitted to two- or three-exponential functions and extrapolated to the beginning of the prepulse segment to determine overall tail amplitude; *n* = 4–6, error bars represent ±SEM. **(C and D)** Dependency of the fast inactivation of wild type and mutant KCNQ1 channels on the duration of activating pulse. **(C)** Exemplary current traces of KCNQ1 wild type and mutant channels elicited by protocol with various duration of activation at +60 mV. Interpulse interval was adjusted to obtain maximal overlay of activation components. **(D)** The dependency of the inactivation fraction from the duration of activating pulse; *n* = 3–6, error bars represent ±SEM.

**Figure S3.**
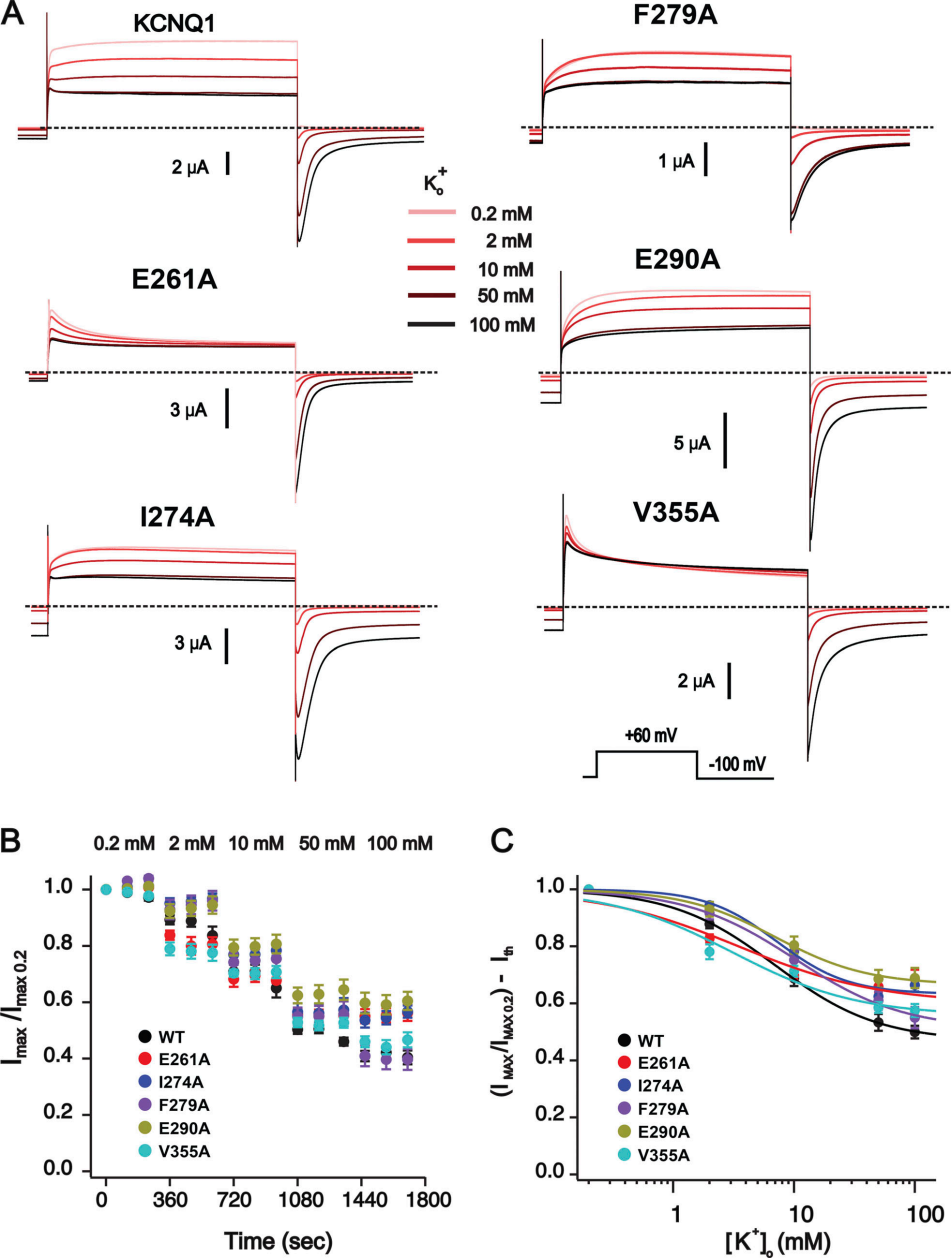
**K**^**+**^_**o**_**-dependent inhibitions of the KCNQ1 mutants exhibiting fast inactivation of various degrees. (A)** Sample current traces of mutant KCNQ1 channels at the five different K^+^ concentrations indicated. Dashed lines correspond to 0 current. **(B)** *I*_max_ values for channels shown in A normalized to the initial point at 0.2 mM K^+^_o_. *n* = 4–6, error bars indicate ±SEM. **(C)** The dose–response curves of the channels shown in A after correction of data for theoretical GHK values assuming [K^+^]_in_ = 109 mM (Weber, 1999). Solid lines represent the fit of the data to the Hill equation, *n* = 4–6, error bars indicate ±SEM.

**Figure S4.**
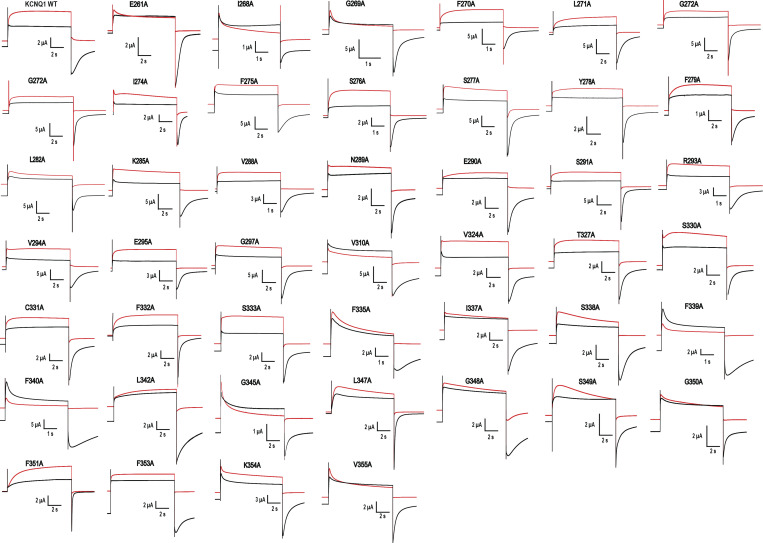
**The external K**^**+**^** sensitivity of alanine mutants of the pore region (amino acids I268–V355).** Typical current traces of alanine mutants measured at 0.2 mM (red and 100 mM (black) external K^+^. Currents were elicited by depolarization of membrane from holding potential −100 to +60 mV. Recordings were started at 0.2 mM K^+^_o_. After stabilization of the amplitudes, the 100 mM K^+^ containing solution was applied. Red traces correspond to the last recording point in 0.2 mM K^+^, black traces correspond to the first point in 100 mM K^+^.

**Figure S5.**
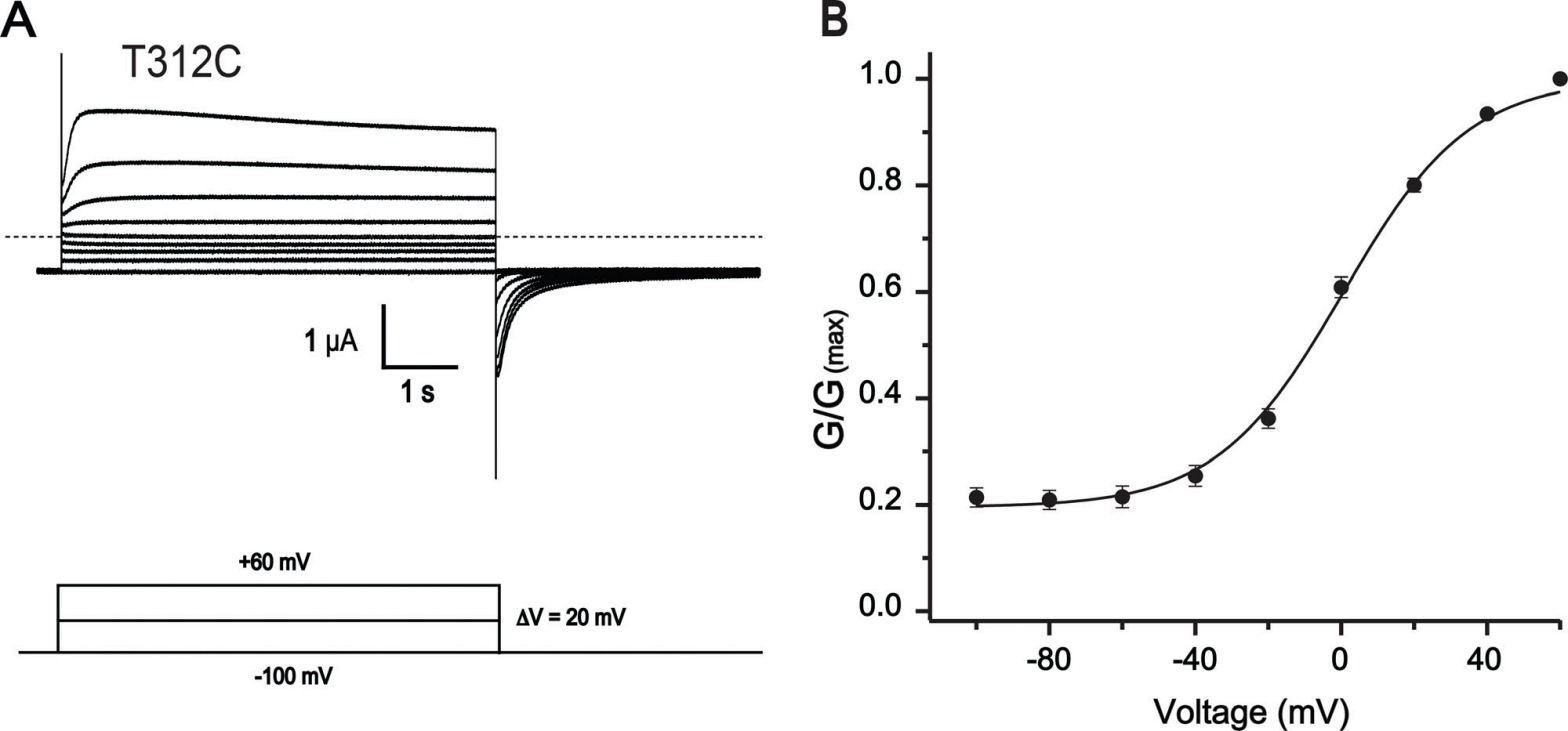
**Voltage-dependent activation of KCNQ1 T312C mutant. (A)** Exemplary current traces of the T312C mutant in response to stepwise depolarizing pulses from a holding potential of −100 mV to +60 mV in 20-mV increments. Dashed line corresponds to 0 current. **(B)** Conductance–voltage relationship of the T312C mutant after correction for inactivation. Tail currents were fitted with a double-exponential function and extrapolated back to the beginning of the segment. Values were normalized to current at +60 mV. The average leak currents, estimated in parallel batches of oocytes, were subtracted. Solid curve represents the fit of the data to a Boltzmann function of the form G/Gmax = (1 − Gmin)/{1 + exp[(V1/2 − V)ZF/RT]} + Gmin, where Gmin is fraction of the voltage-independent conductance component given by the horizontal asymptote of the function. Z, F, R, T, and V1/2 have their usual meanings. Gmin = 0.20 ± 0.04, V1/2 = −1.56 ± 0.14, *n* = 8, error bars represent ±SEM.

**Figure S6.**
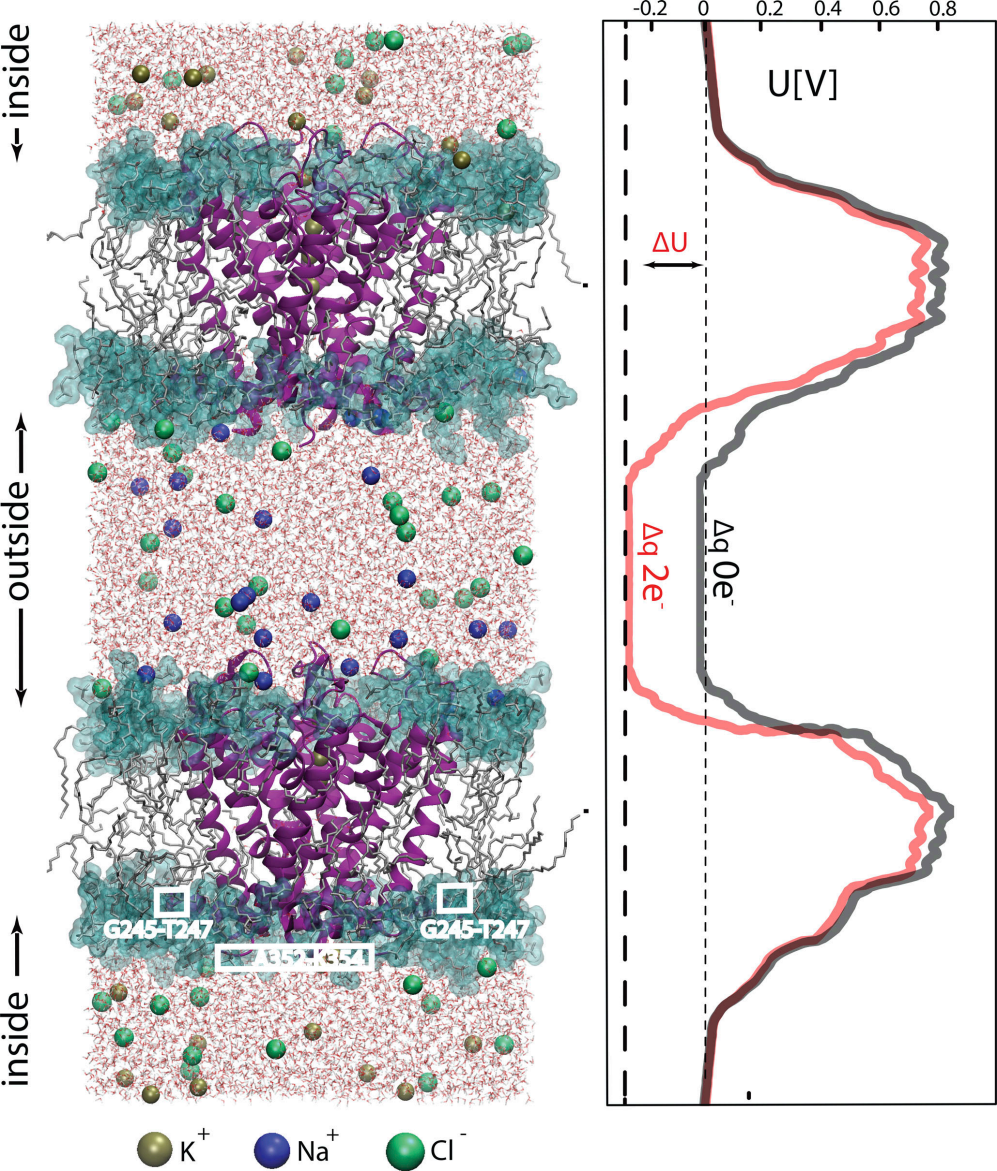
**Double-membrane set-up (**Kutzner et al., 2011**) for simulation of K**^**+**^ **permeation through KCNQ1.** Snapshot of simulation at 5 mM external and 150 internal K^+^ conditions (left). Channels were embedded in two lipid bilayers composed of POPC lipids (dark green) in parallel orientation. Backbone atoms of residues shown white in all subunits were constrained by force constant 1,000 kJ mol^−1^/nm^−2^. Water molecules are shown in red-white. An averaged electric potential profile during this particular MD simulation is represented on the right side. Permeations and dynamics of the lower channel, corresponding to the channel conducting outward current is analyzed.

**Figure S7.**
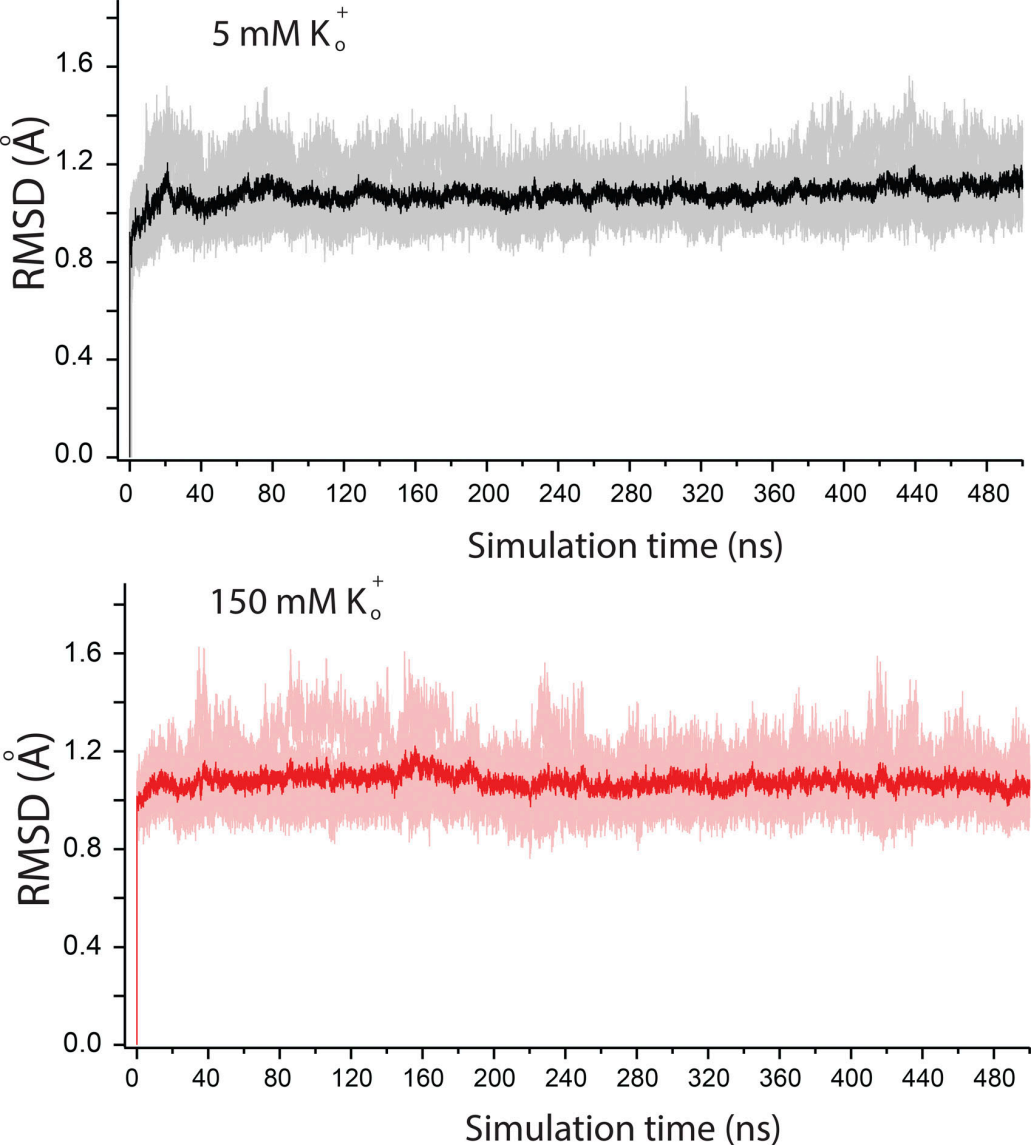
**The RMSD values of protein Cα atoms corresponding to 10 separate simulations for each K**^**+**^_**o**_ **condition (overlaied and shaded).** The average values are indicated by solid lines.

**Figure S8.**
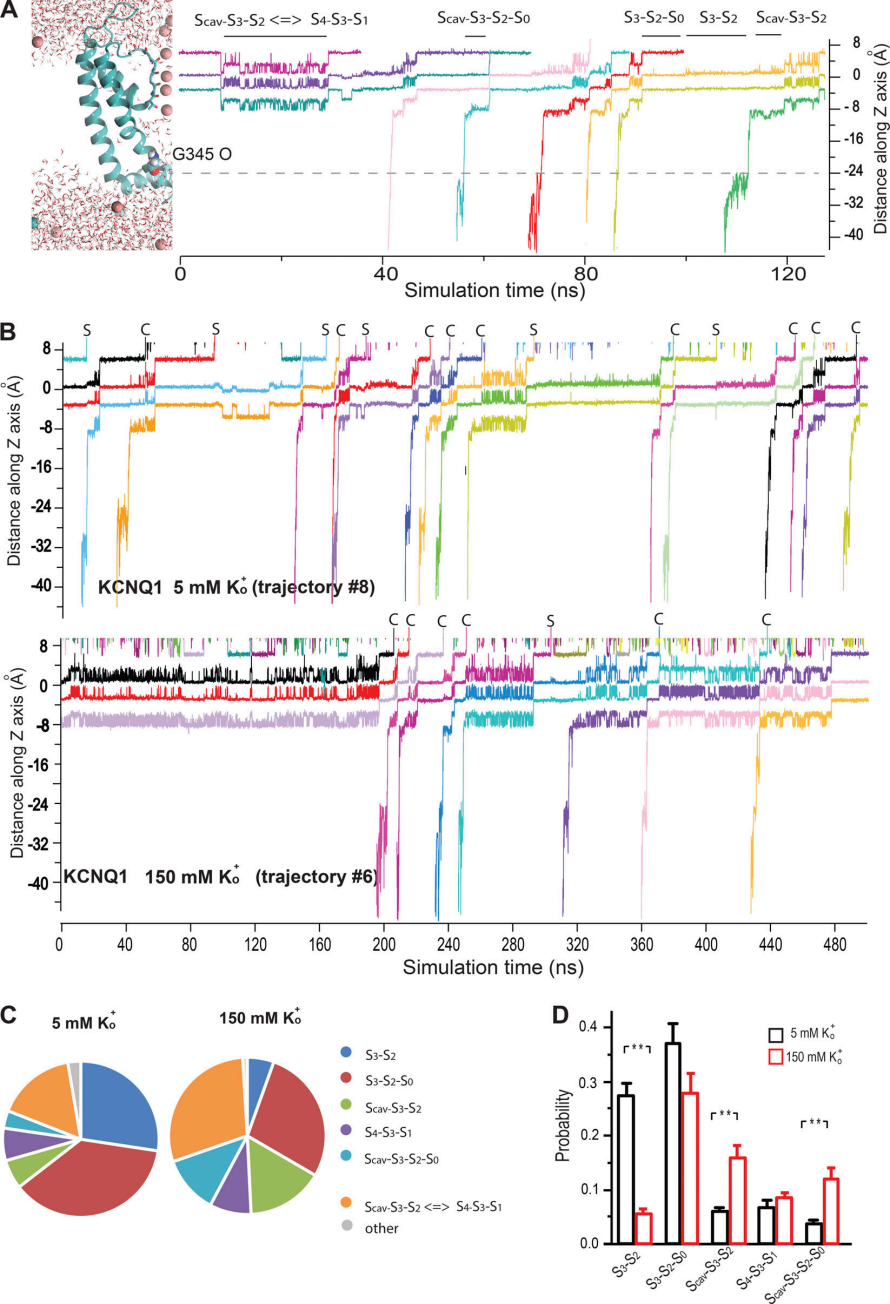
**SF substates and the movement of the ions through the conduction pathway of KCNQ1. (A)** Definition of the SF substates used in this study based on the distance of permeating K^+^ ions from the center of mass of four carbonyl carbons of G314 residues. Frequently observed occupancy substates of the SF are shown above the diagram. Scav, K^+^ binding site in the cavity. **(B)** Sample of diagrams showing K^+^ movement through the SF of the channel at two different K^+^_o_ concentrations investigated. Definitions and symbols are the same as in A. S denotes the spontaneous release of K^+^, C denotes the permeations according to the canonical mechanism (K^+^_in_-induced release). Each colored line represents the movement trajectory of one K^+^ ion. Ion trajectories in the internal medium are removed for clarity. **(C)** Summary of the SF substate distribution at two different concentrations of K^+^_o_ as indicated. **(D)** Statistical analyses of probability of key SF substate occupancies at 5 and 150 mM K^+^_o_ conditions. Error bars in the graphs represent ± SEM; S3-S2 (P = 0.00121); S_cav_-S_3_-S_2_ (P = 0.0013); S_cav_-S_3_-S_2_ (P = 0.00217); n.s., not significant. Student’s unpaired *t* test was used to compare the groups.

**Figure S9.**
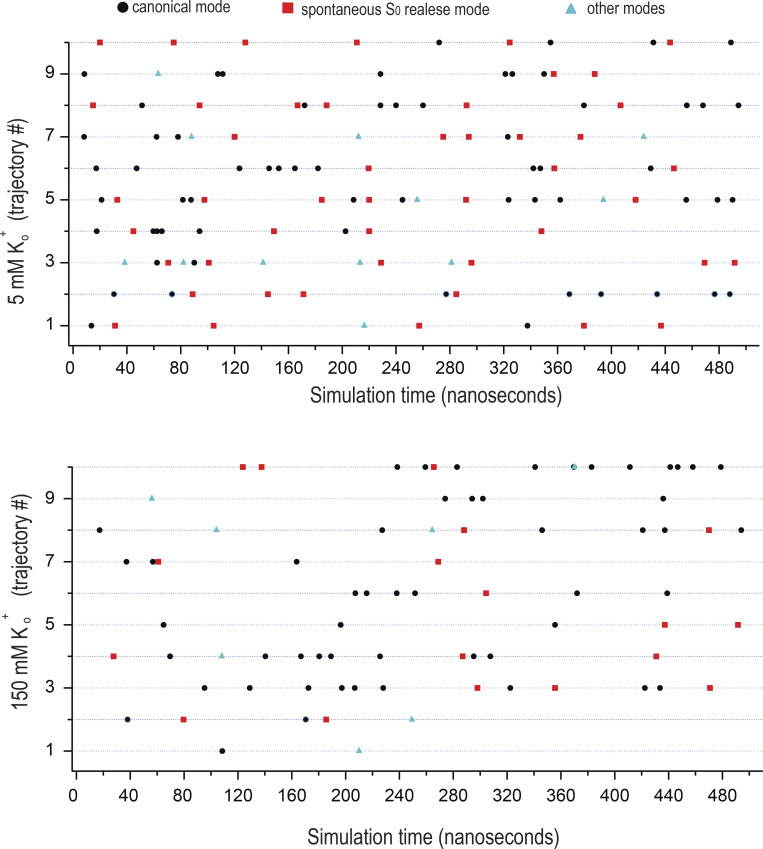
**Interchange of the SF conductive modes at 5 and 150 mM external K**^**+**^ **conditions.** Diagrams show the sequence of ion permeation events over the course of individual MD simulations. The passage of potassium ions was considered as complete and marked on the diagram when the distance between the center of corresponding K^+^ atom in the selectivity filter and the center of mass of four G316 oxygen atoms exceeded 4 Å.

**Figure S10.**
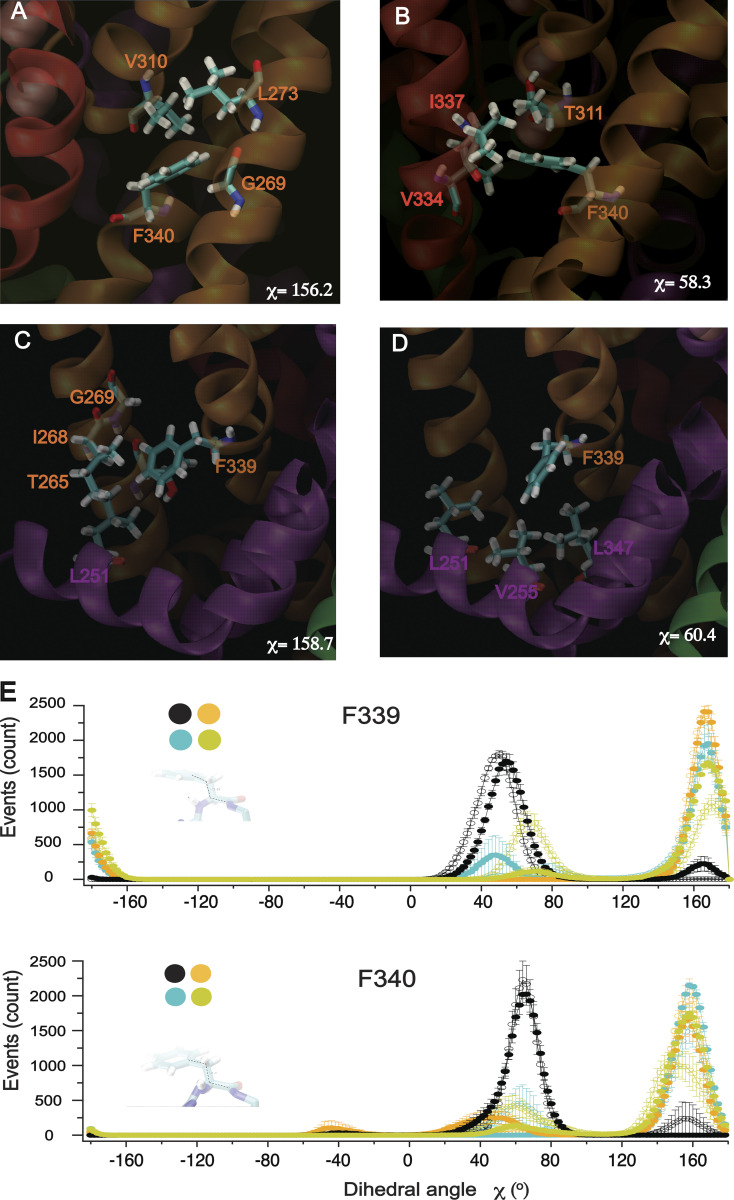
**Two main configurations for the aromatic rings of F339 and F340 residues. (A)** A snapshot from simulation at 5 mM external K^+^ conditions showing the interaction of the F340 side-chain with V310, L273, and G269 residues of the same subunit at a dihedral angle of C-Cα-Cβ-Cγ ∼160^o^. **(B)** Side chains of T311 of the same subunit and those of V334 and I337 of the neighboring subunit stabilize the aromatic ring of F340 after its flipping (dihedral angle of ∼55^o^). **(C)** A snapshot from simulations at 5 mM external K^+^ showing the interaction of F339 with I268, T265, and G269 residues of the same subunit and with L251′ of the neighboring subunit. These interactions stabilize the benzene ring of F339 at C-Cα-Cβ-Cγ dihedral angle of ∼158^o^. **(D)** Molecular interactions with the aliphatic side-chains of L251′, V255, and L347 when the benzene ring of F339 flips (dihedral angle of ∼158^o^). **(E)** Mean amplitude histogram showing the distribution of the dihedral angle of the aromatic rings of F339 and F340 residues. The results of 10 simulations at 5 mM (open circles) and 150 mM K^+^_o_ conditions (filled circles) are shown. Colors of lines and symbols correspond to subunit positions as shown in the inset. Error bars in the graphs represent ± SEM.

**Figure S11.**
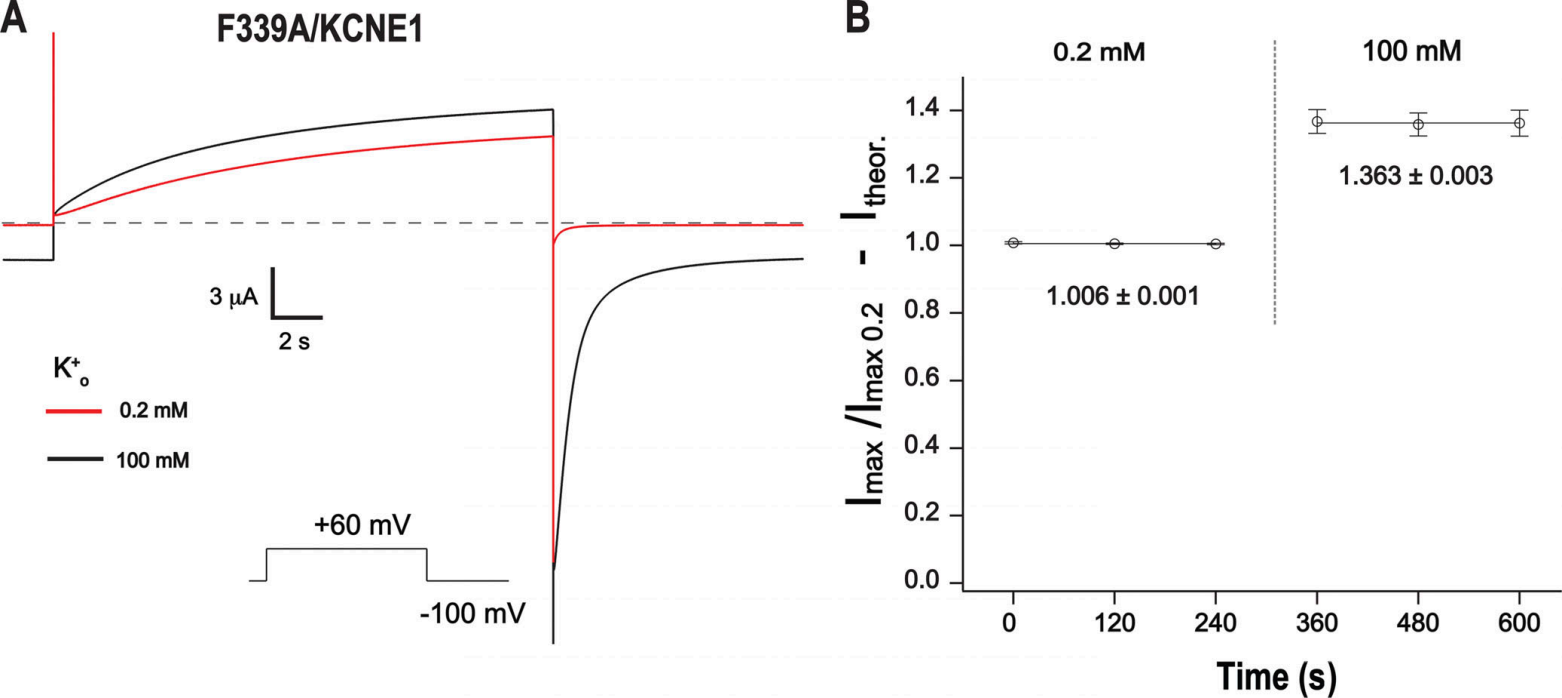
**Potentiation of F339A/KCNE1 heteromeric channel by external K**^**+**^**. (A)** Representative current traces of the F339A/E1 channel recorded at 0.2 and 100 mM external K^+^ conditions. Heteromeric channels were expressed in a 1:1 molecular ratio of α to β subunits. The activation pulse was prolonged to 20 s to reach a steady state. **(B)** Analysis of [K^+^]_o_-dependent inhibition. Date point corresponding to each K^+^_o_ condition were fitted to a linear function. Parameters are shown on the graph; *n* = 6, error bars represent ±SEM.

Fig. S1 illustrates the methodology used to estimate the fractional fast inactivation of KCNQ1 channels. Fig. S2 shows voltage dependency as well as the time dependency of the fast inactivation for wild type and mutant homomeric channels. Fig. S3 presents the magnitude of inhibition of KCNQ1 channels exhibiting various degrees of fractional fast inactivation. Fig. S4 shows exemplary current traces of alanine mutants in the pore region measured at 0.2 and 100 mM external K^+^ conditions. Fig. S5 demonstrates sample current traces of the T312C selectivity-filter mutant and its conductance–voltage relationship. Fig. S6 schematically illustrates the molecular system for the simulation of permeation through the KCNQ1 pore. It also shows the average transmembrane voltage in such a system during simulations. Fig. S7 demonstrates the stability of the protein in this molecular system during the simulation both in symmetrical and asymmetric external K^+^ concentrations. Fig. S8 shows typical K^+^ occupancy substates of the selectivity filter, as well as the distribution of these substates in simulations at 5 and 150 mM external K^+^ conditions. Typical ion movements through the channel pore are illustrated in this figure as well. Fig. S9 shows the interchange of the conductive modes of the selectivity filter during 500 ns simulation at two different K^+^_o_ concentrations. Fig. S10 demonstrates the existence of two main configurations for the aromatic rings of F339 and F340 residues and the statistical distribution among these configurations during simulations. Fig. S11 demonstrates the independence of the potentiating effect K^+^_o_ on the F339A mutant from its slow inactivation phenotype. Videos 1 and 2 visualize the permeations occurring according to the canonical and spontaneous S_0_ modes, respectively. Video 3 shows the delay of forward K^+^ transmissions in the selectivity filter of the channel at high external K^+^ conditions. In Video 4, the flipping of aromatic rings of F339 and F340 residues is visualized.

## Results

### K^+^_o_-sensitivity of KCNQ1 mutants with markedly reduced inactivation

**Figure 1.**
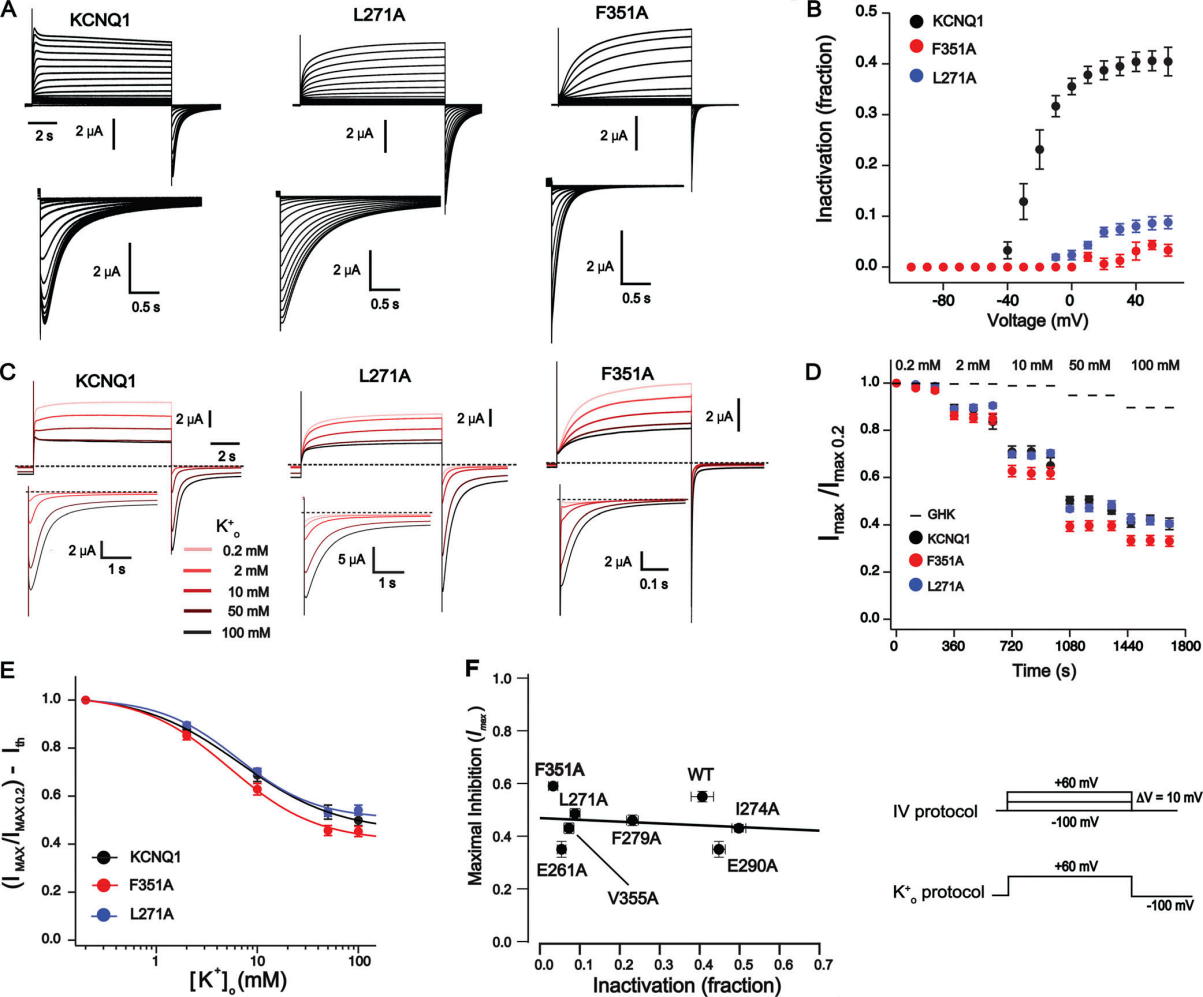
**[K**^**+**^**]**_**o**_**-dependent inhibition of mutant KCNQ1 channels with altered inactivation. (A)** Typical current traces of KCNQ1 channels in 20 mM [K^+^]_o_. Below IV traces the tail currents are shown enlarged to visualize inactivation. **(B)** The extent of the fast inactivation depends on prepulse voltage. The y axis represents the fraction of inactivated channels, *n* = 4–10; error bars represent ±SEM. **(C)** Inhibition of wild type and mutant KCNQ1 channels by K^+^_o_ shown in A. Dashed line represents 0 current. Tail currents recorded in various [K^+^]_o_ are shown enlarged. **(D)** Normalized mean peak currents over three continuous time points at five different [K^+^]_o_ as indicated; *n* = 4–10, error bars represent ±SEM. **(E)** Concentration–response curves after correction of data are shown in D for theoretical values. Solid curves represent fits of the data to the Hill equation. Parameters of inhibition are shown in Table 1; *n* = 4–10, error bars represent ±SEM. **(F)** Correlation plot showing the relationship of [K^+^]_o_-induced inhibition of channels to their fractional inactivation at +60 mV. The y axis represents maximal inhibition obtained by fitting the Hill equation to the data (see Table 1). Detailed analysis for five other mutants is shown in Figs. S2 and S3. Solid line represents fit of data to linear function. Pearson correlation coefficient is −0.22, *n* = 4–10, error bars represent ±SEM.

**Figure 2.**
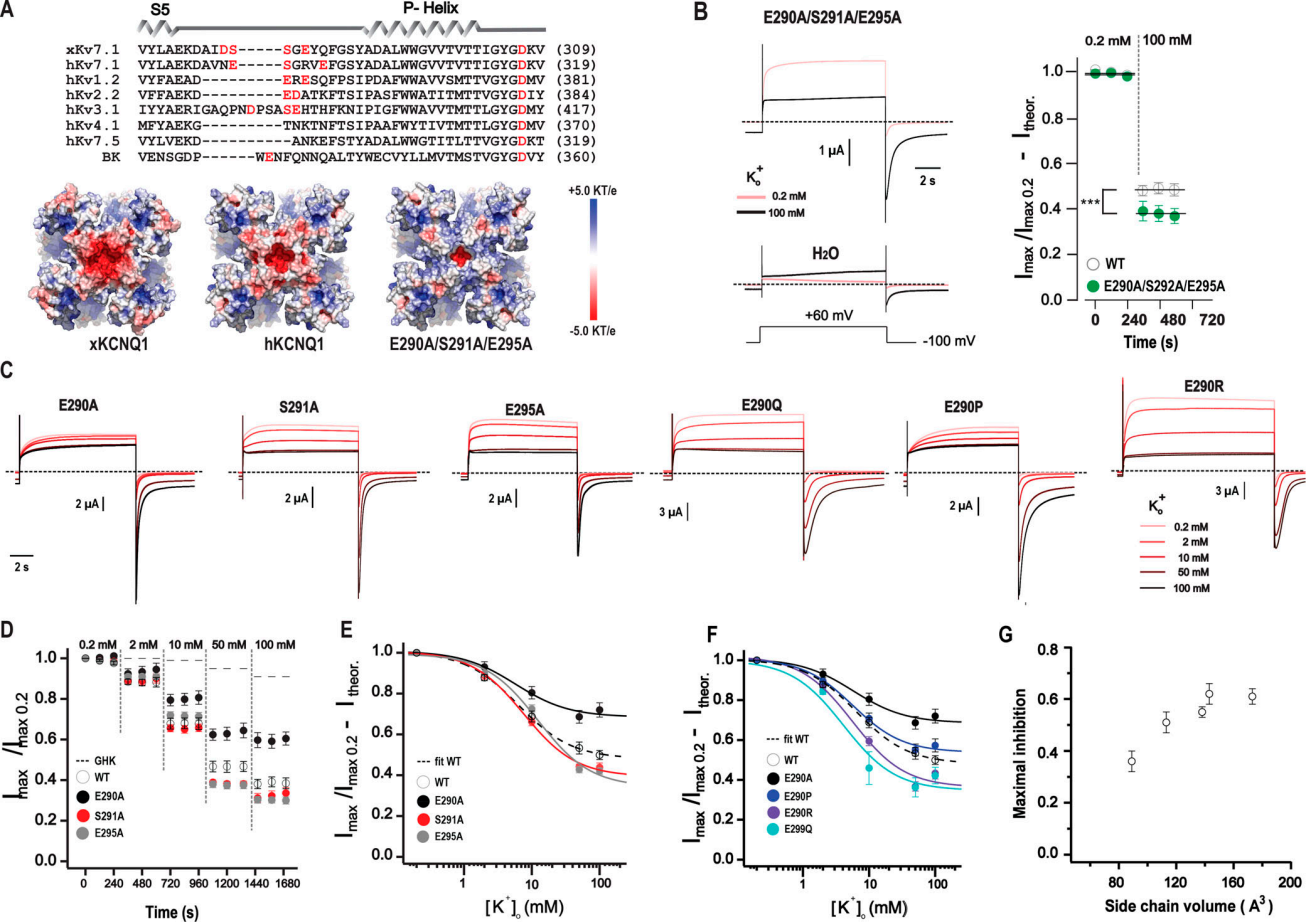
**External electronegative surface of human KCNQ1 plays a minor role in the K**^**+**^_**o**_ **sensitivity of the channel. (A)** Alignment of human and *Xenopus* KCNQ1 sequences along with several other K^+^ channels in S5—pore helix region: *Xenopus* KCNQ1 (xKCNQ1; NCBI ID NP_001116347.1), human KCNQ1 (NCBI ID NM_000218.2). Red letters indicate negatively charged residues making up the externally charged surface. Diagram above the sequences corresponds to secondary structure elements corresponding to the human KCNQ1 structure (Sun and MacKinnon, 2020). Below is a representation of the surface potential of wild type KCNQ1 channels and that of the modeled human triple mutant scaled from −5 KT/e to 5 KT/e (top view). **(B)** Representative current traces and analysis of [K^+^]_o_-dependent inhibition of the triple mutant compared to wild type. Parameters of the linear fits are as follows: WT, 0.2 mM K^+^ = 0.98 ± 0.02 and 100 mM K^+^_o_ = 0.47 ± 0.02; 290A/291A/295A mutant, 0.2 mM K^+^ = 0.98 ± 0.02 and 100 mM K^+^ = 0.38 ± 0.03, *n* = 8, error bars represent ±SEM; ***P = 0.0007 ANCOVA. **(C)** Sample current traces of single mutants at five different [K^+^]_o_ conditions as indicated. **(D)** Peak current amplitudes in variable [K^+^]_o_ normalized to the first point of recording at 0.2 mM [K^+^]_o_ for E290A, S291A, and E295A channels, *n* = 5–9, error bars represent ±SEM. **(E and F)** Concentration–response curves for mutants after correction for theoretical reduction (Ith) assuming [K^+^]_in_ = 108 mM (Weber, 1999). Solid lines are fits of the Hill equation to data. Dashed line represents fit of wild type data. Parameters of fits are shown in Table 1. **(G)** Dependency of maximal inhibition from side-chain volume at E290 position. Values of maximal inhibition are taken from Table 1. *n* = 4–6, error bars represent ±SEM.

We initially investigated the influence of different [K^+^]_o_ on outward current mediated by homomeric KCNQ1. In agreement with previous studies (Yang and Sigworth, 1998; Larsen et al., 2011; Wang et al., 2015), we observed a significant reduction in KCNQ1-mediated current at +60 mV in the range of [K^+^]_o_ between 0.2 and 100 mM (Fig. 1). Fitting a Hill equation to GHK-corrected data (Fig. 1 E) resulted in inhibition parameters (Table 1) comparable to what was reported in earlier studies in *Xenopus* oocytes and CHO cells (Larsen et al., 2011; Wang et al., 2015). Activated homomeric KCNQ1 channels undergo a fast inactivation process also known as hook inactivation (Sanguinetti et al., 1996; Pusch et al., 1998; Tristani-Firouzi and Sanguinetti, 1998). The name originates from the hook-like shaped tail current at repolarization potentials (−100 mV in Fig. 1 A), which reflects two simultaneously ongoing processes: (1) the recovery of channels from inactivation and (2) channel deactivation. We next tested the hypothesis of whether K^+^_o_ sensitivity of KCNQ1 is associated with the fast inactivation by investigating the F351A mutant previously shown to exhibit considerable deficiency in inactivation (Hou et al., 2017) and L271A that is located in the S5 segment and markedly reduces the inactivation as well (unpublished data). As an initial step, we quantified inactivation in these mutants using a method (Tristani-Firouzi and Sanguinetti, 1998) described in detail in Fig. S1 depending on (1) the voltage of depolarization (Fig. 1, A and B) and (2) the duration of activating pulses (Fig. S2, C and D). The results show that the voltage- and time-dependent inactivation of L271A and F351A channels is strongly suppressed (Fig. 1, A and B; and Fig. S2 D). Analysis of K^+^_o_ dependency in these mutants revealed that they are inhibited by K^+^_o_ to an extent comparable with the wild type (Fig. 1, C–E; and Table 1). We also studied several other mutants showing different degrees of inactivation (Fig. S2, Fig. S3, and Table 1), including I274A, which exhibits enhanced inactivation, and performed correlation analysis represented in Fig. 1 F. Results clearly demonstrate that K^+^_o_-dependent inhibition of KCNQ1 is not correlated with the extent of fast inactivation.

**Table 1.** Parameters of KCNQ1 inhibition by external K^+^ determined via fitting the Hill equation to the data (see Materials and methods)

Channel	IC_50_ (mM)	*I*_max_	nH	*n*
Q1 WT	7.67 ± 1.61	0.55 ± 0.02	1.02 ± 0.19	8
Q1+E1	11.42 ± 4.27	0.24 ± 0.04***	0.87 ± 0.14	9
EQQ	13.40 ± 4.72	0.44 ± 0.03**	0.91 ± 0.23	6
Q1+E2	16.15 ± 7.62	0.13 ± 0.07***	0.88 ± 0.17	5
Q1+E3	17.7 ± 6.26*	0.54 ± 0.05	0.93 ± 0.09	8
E261A	3.47 ± 1.48*	0.35 ± 0.04**	0.77 ± 0.26	4
L271A	7.01 ± 0.57	0.53 ± 0.02	1.17 ± 0.19	4
I274A	7.24 ± 1.31	0.41 ± 0.03**	1.41 ± 0.39	9
F279A	10.3 ± 2.31	0.47 ± 0.02	0.96 ± 0.14	5
E290A	7.01 ± 1.81	0.36 ± 0.04**	1.13 ± 0.18	6
E290P	6.71 ± 1.17	0.51 ± 0.04	1.07 ± 0.24	5
E290R	5.98 ± 0.79	0.61 ± 0.03**	1.04 ± 0.15	5
E290Q	4.03 ± 1.12*	0.62 ± 0.04**	1.02 ± 0.31	4
S291A	9.14 ± 3.98	0.63 ± 0.05*	0.89 ± 0.39	6
E295A	15.12 ± 5.91	0.71 ± 0.04**	0.92 ± 0.16	9
F335A	1.19 ± 0.20*	0.17 ± 0.04***	0.91 ± 0.33	4
F351A	5.64 ± 0.73	0.58 ± 0.02	1.10 ± 0.13	10
F351A Rb^+^	1.81 ± 0.17***^#^	0.71 ± 0.02***#	1.16 ± 0.09	8
V355A	3.12 ± 1.89	0.44 ± 0.07	0.87 ± 0.28	4

IC_50_, concentration of half-maximal inhibition; *I*_max_, the maximal inhibition; nH, the Hill coefficient. Data are represented as mean ± SEM. One-way ANOVA, Dunnett’s post-hoc test, *P < 0.05, **P < 0.01, ***P < 0.001; ^#^, Student’s *t* test; comparison group is F351A for K^+^_o_ conditions.

### Negative charge in the turret region plays no principal role

The recently resolved atomic structure of *Xenopus* KCNQ1 revealed a unique negatively charged extracellular protein surface (Fig. 2 A) surrounding the pore entrance of the channel (Sun and MacKinnon, 2017). Calculation of the electrostatic potential based on recent cryo-EM structures of KCNQ1 channels (Sun and MacKinnon, 2017, 2020) shows less electronegativity in the human ortholog (Fig. 2 A). Nevertheless, negatively charged side chains forming the surface of human KCNQ1, hypothetically, could account for K^+^_o_ sensitivity by attracting K^+^ from the external medium. Such a postulation is in accordance with an earlier report showing a reduction in K^+^_o_ sensitivity by mutation of the E290 residue (Wang et al., 2015). To understand more about the impact of an external negative potential on the inhibitory effects of K^+^_o_, we first generated a mutant in which all amino acids involved in the formation of the electronegative surface (E290, S291, E295, and D317) were exchanged for alanine. KCNQ1-specific current could be recorded neither from *Xenopus* oocytes injected with the corresponding mRNA nor from transfected HEK 293T cells (data not shown). The aspartic acid at the 317 position is strongly conserved among K^+^ channels (Fig. 2 A) suggesting that this residue is unlikely to be responsible for K^+^_o_ sensitivity of the channel. Thus, we next investigated the E290A/S291A/E295A triple mutant. Marked reduction of current expression hampered the analysis of the concentration dependency in this channel. Nevertheless, the comparison of current amplitudes at 0.2 mM and 100 mM K^+^_o_ concentrations demonstrated that K^+^_o_ strongly inhibits the channel, the extent of which was even slightly (∼8%) higher than that of the wild type (Fig. 2 B). We next assessed the K^+^_o_ sensitivity of corresponding single alanine mutants as well. The extent of inhibition of S291A and E295A mutants by external K^+^ was very similar to that of the triple mutant when current amplitudes at 0.2 mM and 100 mM K^+^_o_ are compared (Fig. 2, C–E). The E290A mutation induced about 34% reduction in K^+^_o_-dependent inhibition relative to wild type (Fig. 2, C–E; and Table 1), which was consistent with an earlier study conducted in CHO cells with E290C mutant (Wang et al., 2015). We next introduced a positive charge at the E290 position by exchanging glutamic acid for arginine. The resultant channel exhibited marginally (∼6%) increased sensitivity to external K^+^ (Fig. 2, C and F). Neutralization of the E290 side chain by E290Q mutation, which preserves the side chain volume, produced a channel with K^+^_o_ sensitivity similar to E290R, additionally causing a small leftward shift (ΔIC_50_ = 3.64 ± 1.96 mM) in the concentration–response curve (Fig. 2, C and F; and Table 1). Proline substitution of the E290 position created a channel with K^+^_o_ sensitivity very similar to wild type (Fig. 2, C and F; and Table 1). These results suggest that the negative charge at the E290 position is not necessary for K^+^_o_ sensitivity. The side-chain volume at the E290 position seems to be positively correlated with the extent of K^+^_o_-induced inhibition (Fig. 2 G). Remarkably, the E290W mutant with the largest possible side chain volume did not produce functional channels (data not shown). *Xenopus* oocytes injected with mRNA encoding the D317A mutant produced small currents that were not suitable for analysis, and proline, glycine, or tryptophan substitutions at this position did not result in functional expression. Altogether, these results indicate that the electronegative pore surface of KCNQ1 is not the cause of the K^+^_o_ sensitivity of the channel. A slightly increased K^+^_o_ sensitivity in S291A, E295A, E290R, E290Q, and E290A/S291A/E295A mutants as well as its decrease in E290A mutant is consistent with the general picture of the broad mutagenesis study described below, indicating that statistically significant changes in K^+^_o_ sensitivity can be induced by mutations located in various regions of the channel pore.

### Alanine scanning mutagenesis of the pore domain

**Figure 3.**
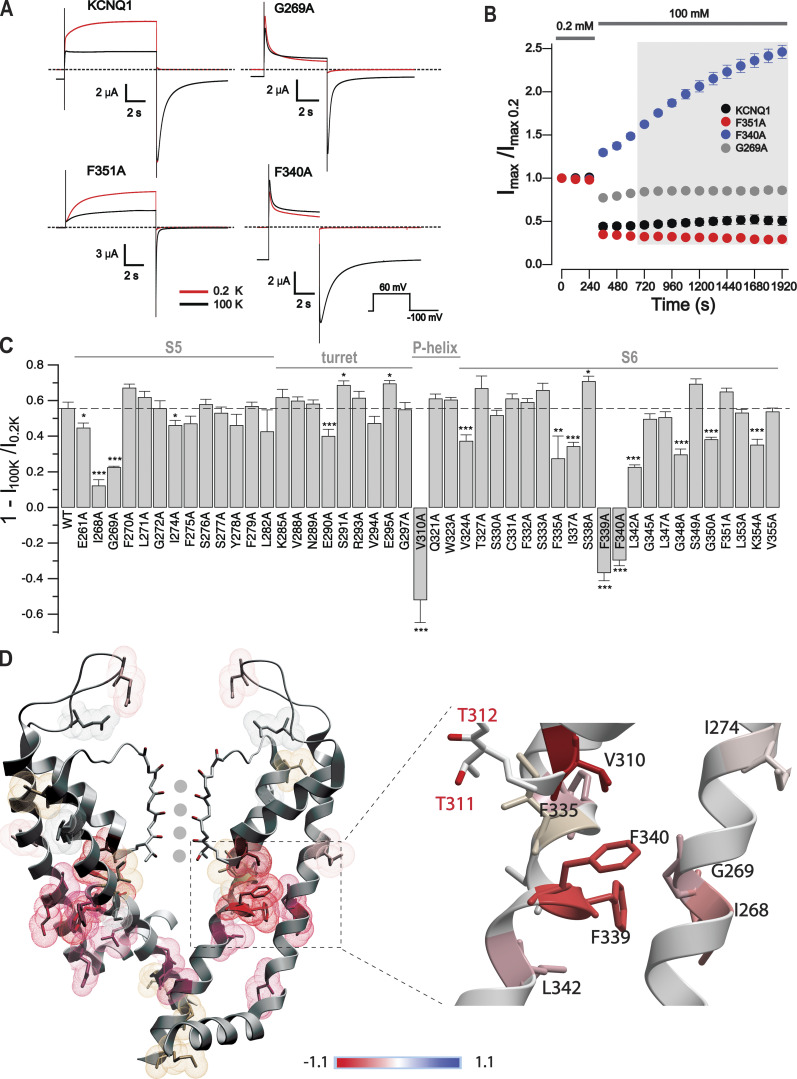
**K**^**+**^_**o**_ **sensitivity of alanine mutants spanning pore residues I268–V355. (A)** Sample current traces from representative KCNQ1 channels in 0.2 mM and 100 mM [K^+^]_o_. Dashed lines show 0 current. **(B)** Peak current analysis for representative channels shown in A normalized to the first point at 0.2 mM K^+^_o_. Prolonged recordings at 100 mM K^+^_o_ is shown, *n* = 4–7, error bars represent ±SEM. **(C)** Summary of current amplitude changes in 100 mM [K^+^]_o_ relative to 0.2 mM [K^+^]_o_ for alanine mutants. *n* = 5–12, error bars represent ±SEM, one-way ANOVA, Dunnett’s post-hoc test, *P < 0.05, **P < 0.01, ***P < 0.001. Alanine mutants yielding no currents in oocytes are not shown. **(D)** Localization of residues, alanine mutation of which exert statistically significant changes compared with wild type on the recent cryo-EM structure of human KCNQ1 (PDB ID: 6UZZ; Sun and MacKinnon, 2020). The extent of mutation-induced changes, represented as color-coded arbitrary intensity, is calculated as ΔI_rel_ = [1 − I_100K_ Mut/I_0.2K_ Mut] – [1 − I_100K_ WT/I_0.2K_ WT]. Scale bar indicates ΔIrel. Inset shows a closer view of the region with a strong mutational effect.

We next employed alanine scanning mutagenesis to map residues that are essential for the K^+^_o_ sensitivity of KCNQ1. Since no considerable voltage-dependency is observed for the K^+^-induced KCNQ1 inhibitory process (Larsen et al., 2011), we studied the pore residues spanning the I268–V355 segment with a simplified approach that compares current at 0.2 and 100 mM K^+^_o_ conditions (Fig. 3, A–C; and Fig. S4). This approach is reasonable due to our finding that IC_50_ values are not considerably changed for most of the mutants studied (Table 1). For more than half of the alanine mutants, only minor alterations were observed compared with the wild type (Fig. 3 C). 17 mutations induced statistically significant changes in K^+^_o_ sensitivity to variable extents. Of those, V310A, F339A, and F340A exhibited a noticeable current potentiation by high K^+^_o_ (Fig. 3, A–C; and Fig. S4). Mapping mutations that induced statistically significant changes onto the recent KCNQ1 structure revealed that the moderate changes are inducible by mutations located in different parts of the channel pore (Fig. 3 D). Stronger alterations in K^+^_o_ sensitivity are induced by mutations of residues that are buried deeply in the hydrophobic part of the channel that is not accessible to external K^+^ (Sun and MacKinnon, 2020). Congregation of these residues below the SF (Fig. 3 D) pointed toward the potential involvement of the SF in the modulation process.

### External permeant ions induce current modulation

One possible explanation for the involvement of the SF is that the enhanced binding of the K^+^_o_ to its extracellularly located site may trigger functional modulations in SF leading to current inhibition. To ascertain that the inhibition is caused by enhanced binding of K^+^_o_ to channel but not due to the reduction of [Na^+^]_o_ necessary to keep the ionic strength of the external medium, we next studied the comparative effect of the external permeant (Rb^+^, K^+^) versus non-permeant (Na^+^, NMDG^+^) cations in representative mutant channels. We selected one channel in each mutant category—F351A, G269A, and F340A—that exhibited wild type-like, a weaker-, and a “reverse” K^+^_o_ sensitivity, respectively, to conduct these experiments. If inhibition is triggered by the binding of permeant ions to SF, these experiments had the potential to provide insights into the mechanism of the inhibition process since K^+^ channels exhibit differential functional properties depending on whether K^+^ or Rb^+^ is bound to their SFs.

**Figure 4.**
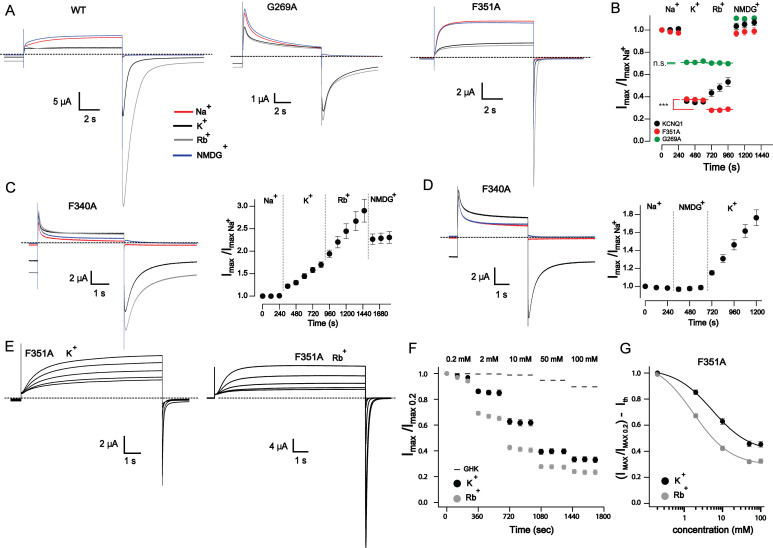
**Inhibition of outward KCNQ1 current is induced by external permeant ions. (A)** Sample current traces of KCNQ1 channels at 100 mM external monovalent cations as indicated. Currents were continuously recorded at 120-s interpulse intervals. Representative traces correspond to second point of each type of the monovalent ion shown in B. Dashed lines represent 0 current. **(B)** Changes of outward peak current amplitude at +60 mV upon sequential substitution of external monovalent cations normalized to amplitudes of the first point recorded in external 100 mM Na^+^, *n* = 5–8, error bars represent ±SEM. Lines are the fits of the G269A and F351A data to linear function; F351AK^+^ = 0.374 ± 0.003; F351ARb^+^ = 0.282 ± 0.003; G269AK^+^ = 0.701 ± 0.002; G269ARb^+^ = 0.697 ± 0.003, ***P = 0.0007; n.s., nonsignificant (ANCOVA linear regression analysis). **(C)** Representative current traces from recordings of the F340A mutant in the presence of various external monovalent cations (100 mM). The corresponding analysis is shown on the right side of the traces. *n* = 5, error bars represent ±SEM. **(D)** Current traces and the corresponding analysis of F340A mutant when the sequence of solution exchange was modified as indicated; *n* = 4, error bars represent ±SEM. **(E)** Example of current traces mediated through F351A mutant at five different concentrations of external K^+^ and Rb^+^ as indicated. **(F)** Peak current inhibition for F351A in external K^+^ and Rb^+^ conditions normalized to their values at 0.2 mM, *n* = 8 for Rb^+^_o_ and *n* = 10 for K^+^_o_, error bars represent ±SEM. **(G)** Concentration–response curves after subtraction of theoretical values according to the GHK equation. Solid curves represent fits of the data to the Hill equation. The parameters of inhibition are shown in Table 1. Error bars represent ± SEM.

The inhibition of wild type KCNQ1 by 100 mM external K^+^ was mimicked by equimolar external Rb^+^ (Fig. 4, A and B). We also detected a minor alteration in the shape of the outward current trace (Fig. 4 A) and a tendency for current to increase during recordings in external Rb^+^ (Fig. 4 B). Such a trend was not observed at high K^+^_o_ conditions even after prolonged K^+^_o_ exposure (Fig. 3 B). We speculate that this effect could be related to the gradual elevation of intracellular Rb^+^ in oocytes due to large inward-holding currents. The continuously increasing intracellular Rb^+^ then would lead to a larger outward current due to higher Rb^+^ conductance of KCNQ1 compared with K^+^ (Pusch et al., 2000). Substitution of external Rb^+^ with the impermeable organic cation NMDG^+^ having a much larger ionic diameter (∼7.3 Å) than Na^+^ (1.9 Å) eliminated inward currents and re-established amplitudes observed at initial Na^+^ conditions (Fig. 4, A and B). Rb^+^_o_ also mimicked the effect of K^+^_o_ on the F351A and G269A mutants with subsequent restoration of current amplitudes observed at starting conditions when NMDG^+^ was applied (Fig. S4, A and B). The G269A mutant did not show a similar trend of increase in outward currents in the external 100 mM Rb^+^ condition despite the holding current in this mutant being comparable with the wild type (Fig. 4 A). This can be explained by a lower Rb^+^/K^+^ conduction ratio of the G269A mutant (see tails in Fig. 4 A). The potentiation of the F340A channel by high K^+^_o_ (Fig. 3, A–C) was slightly augmented by application of external Rb^+^ (Fig. 4 C). Strikingly, in this case, the Rb^+^_o_ substitution by NMDG^+^ did not restore the current amplitudes observed in the initial Na^+^_o_ condition (Fig. 4 C). We did not study this phenomenon in further detail. However, experiments with the application of external NMDG^+^ after Na^+^_o_ clearly demonstrated that NMDG^+^ itself has no influence on F340A (Fig. 4 D). A slight tendency toward increased inhibition in G269A mutant by Rb^+^_o_ compared with K^+^_o_ was not statistically significant (Fig. 4 B). In the F351A mutant, the inhibition of outward current was slightly more pronounced when Rb^+^_o_ was applied**.** This prompted us to investigate the concentration dependency under external Rb^+^ conditions for this mutant. The F351A channel has a very small constitutively open fraction allowing for prolonged recordings at negative potentials (Fig. 4, E and F). The results show that external Rb^+^ is a more effective inhibitor of the outward current than K^+^_o_ (Fig. 4 G and Table 1). Altogether, these results suggest that the modulation of KCNQ1 is induced by permeant ions applied at the extracellular side of the membrane. Observed alterations in the inhibitory effect of Rb^+^_o_ compared with K^+^_o_ most likely reflect the difference in the binding properties of these cations on the external modulatory site of the channel.

### Loss of selectivity and external K^+^ sensitivity in the T312C mutant

**Figure 5.**
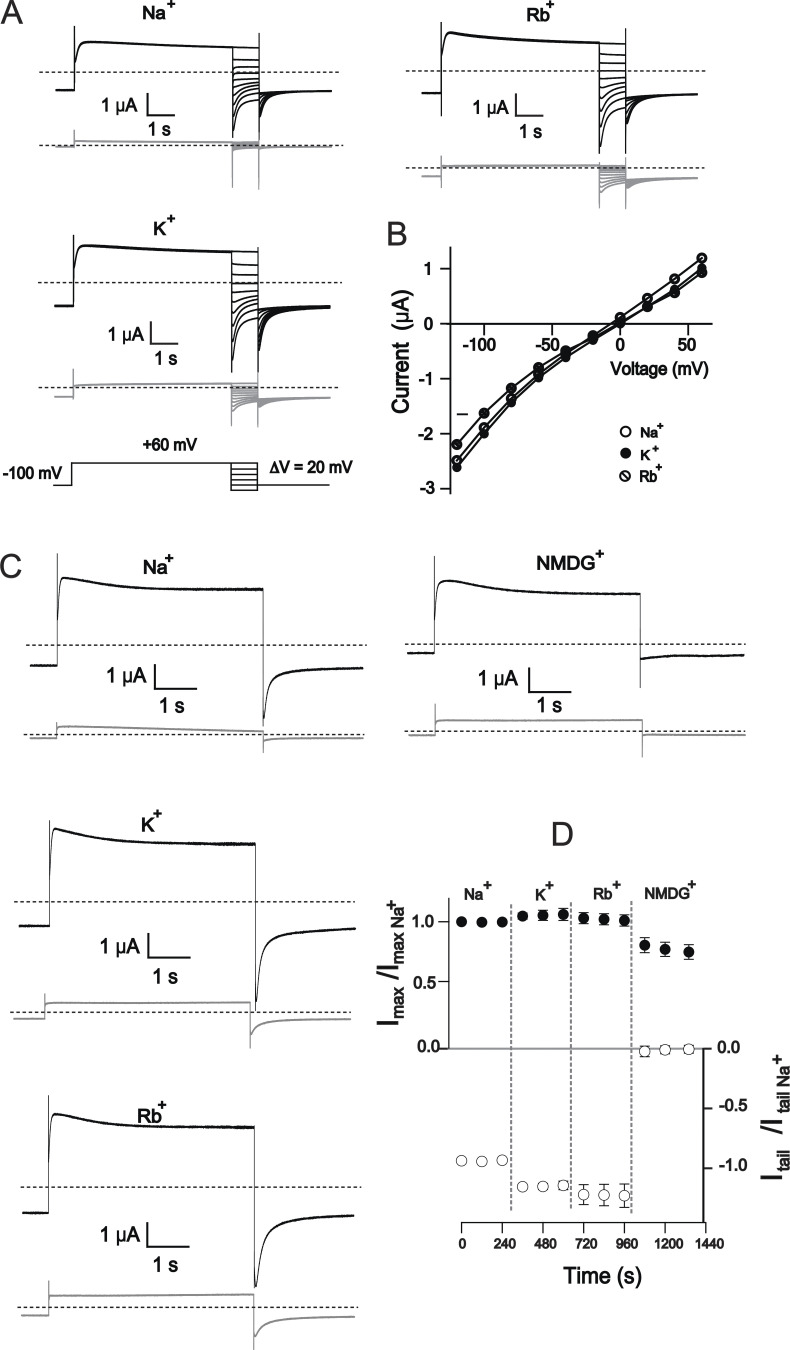
**K**^**+**^_**o**_ **sensitivity of nonselective T312C mutant. (A)** Sample current traces of the T312C mutant recorded in 100 mM external Na^+^, K^+^, and Rb^+^ (black traces) solutions to calculate reversal potential changes. Recordings from water-injected oocytes from the parallel batch under identical conditions are shown in gray. **(B)** Instantaneous current–voltage curves after correction for average leak current from water-injected oocytes. Three separate batches of oocytes were injected with T312C cRNA from two separate syntheses. Results of statistical analysis of reversal potential changes are shown in the text, *n* = 9, error bars represent ±SEM. **(C)** Representative current traces of T312C (black) and the corresponding water-injected oocytes (gray) upon exchange of monovalent cations in extracellular solution as indicated. **(D)** Analysis of peak and tail current amplitudes shown in C normalized to Na^+^. Leak currents were estimated using a parallel batch of oocytes, *n* = 4, error bars represent ± SEM.

To further investigate the involvement of the SF in the process of K^+^_o_ dependency of KCNQ1, we searched for mutation(s) that might induce noticeable alterations in SF function. Although our previous study suggested that most of the mutations in the K^+^-selective motif or its proximity render the KCNQ1 channel nonfunctional (Ma et al., 2011), we found that the T312C mutant retained functional expression and the voltage-gating properties of KCNQ1 (Fig. S5). Membrane potentials near to 0 mV together with large inward tail currents in external K^+^-free solution indicated that the T312C mutation affects the ion selectivity of KCNQ1 (Fig. 5, A and B). Analysis of the relative permeability via estimation of reversal potential for external Na^+^ and K^+^ conditions revealed a dramatic drop of K^+^ selectivity in T312C (P_Na__+_/P_K__+_ = 0.77 ± 0.06, *n* = 9, ±SEM vs. 0.016 for I_ks_; Matsuura et al., 1987). In addition, we observed a significant increase in relative Rb^+^ permeability (P_Rb__+_/P_K__+_ = 0.93 ± 0.02 in T312C, (*n* = 9) vs. 0.75 ± 0.01 in WT, *n* = 5, P = 0.0076, Student’s *t* test; ±SEM). More remarkably, no considerable inhibition of outward current in T312C was observed upon replacement of Na^+^_o_ with equimolar K^+^_o_ or Rb^+^_o_ (Fig. 5, C and D). Hence, the T312C mutation induces a loss of channel selectivity—a feature reflecting functional modifications in the SF—which also eliminates K^+^_o_ sensitivity of the channel. This confirmed our hypothesis that the K^+^_o_ dependency of KCNQ1 is attributable to the SF of the channel.

### Single-channel properties at high external K^+^

**Figure 6.**
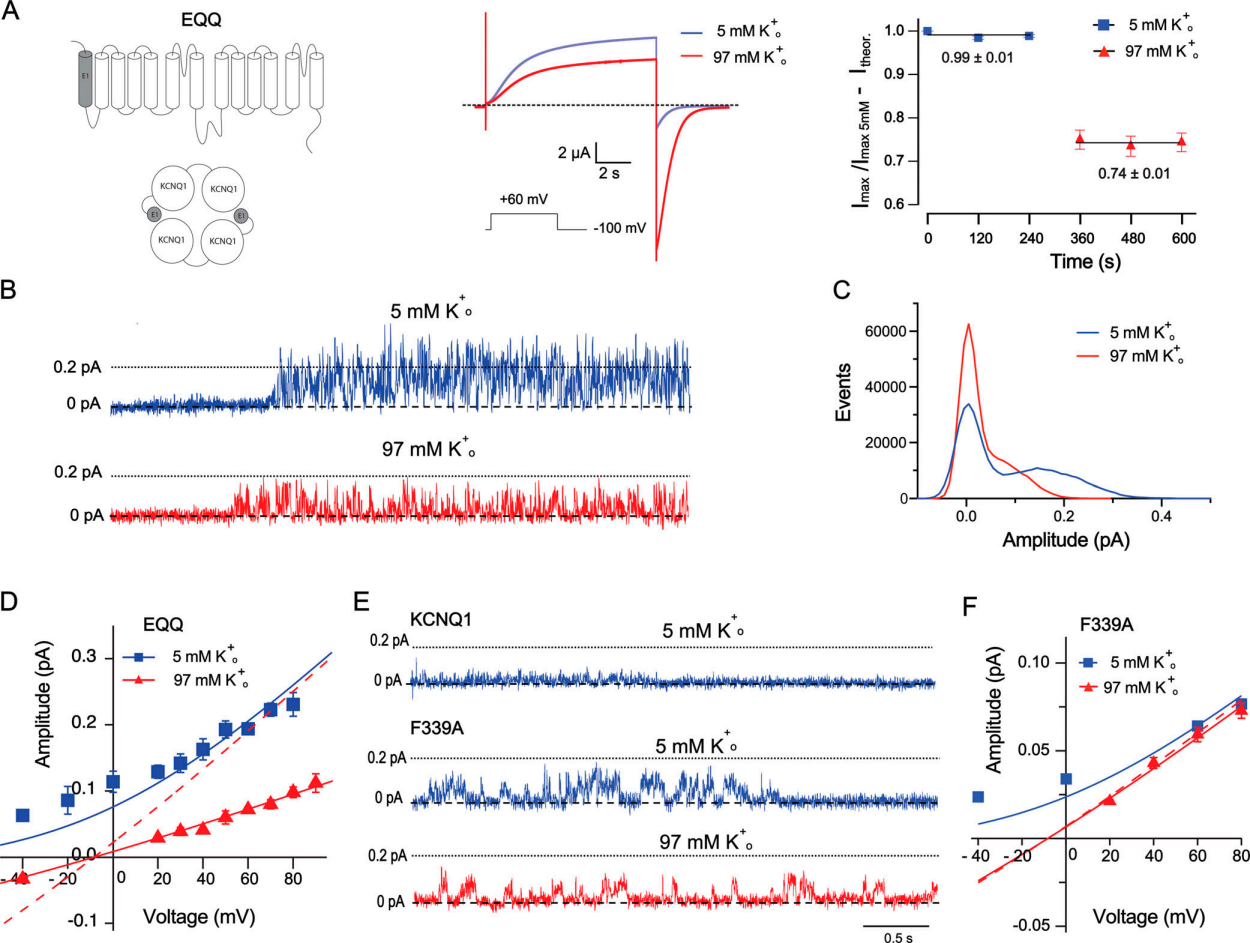
**EQQ single-channel conductance is reduced by high external potassium. (A)** Schematic illustration of EQQ channels (left), representative current traces recorded from oocytes expressing EQQ at 5 and 97 mM K^+^_o_ (middle), and corresponding statistical analysis of peak current at +60 mV (right); *n* = 7, error bars represent ±SEM, traces correspond to linear fit of the data. **(B)** Sample single channel records of EQQ at low and high external K^+^. Cells were pulsed for 4 s to +60 mV from a holding potential of −80 mV. **(C)** All-point histograms of 11 active EQQ single channel sweeps each at 5 mM (blue) and 97 mM (red) external K^+^ with an activation potential of +60 mV. **(D)** Current–voltage data for EQQ single channels at low and high external K^+^. Solid traces correspond to the fits of GHK equation to the data to determine the absolute permeability (P) of the channel, P_5 mM_ = 5.82 × 10^−4^ ± 2.9 × 10^−5^ cm/s; P_97 mM_ = 2.47 × 10^−4^ ± 4.73 × 10^−6^ cm/s (P = 0.013); *n* = 3–9 at each voltage. Dashed curve corresponds to a hypothetical channel without external K^+^ sensitivity. Error bars represent ±SEM. **(E)** Sample current traces from single-channel records of wild type and F339A mutant using identical conditions as for EQQ for 5 mM K^+^ and a holding potential of −10 mV for the 97 mM K^+^_o_ to confirm this was a K^+^ current. **(F)** Current–voltage data for F339A mutant and corresponding fits to GHK equation. Absolute permeability values are as follows: P_5 mM_ = 1.89 × 10^−4^ ± 2.01 × 10^−5^ cm/s; P_97 mM_ = 1.80 × 10^−4^ ± 3.13 × 10^−6^ cm/s (P = 0.038); *n* = 3–4 at each voltage. The theoretical curve for 97 mM K^+^ corresponding to GHK is shown dashed.

To explore the K^+^_o_ dependency of KCNQ1 at the single-channel level, we next recorded EQQ tandem channels expressed in *ltk*-mouse fibroblast cells. EQQ was constructed by sequential linkage of one KCNE1 auxiliary subunit to two KCNQ1 pore-forming subunits (Fig. 6 A) via long flexible linkers (Murray et al., 2016). Expression of EQQ in heterologous systems produces KCNQ1/KCNE1 heteromeric channels with 4:2 stoichiometry (Fig. 6 A; Murray et al., 2016). The choice of EQQ was a compromise between the KCNQ1 homomers with high K^+^_o_ dependency but with a single-channel conductance close to the limit of the resolution of the recordings system (Werry et al., 2013; Hou et al., 2017), and KCNQ1/KCNE1 with much larger conductance (3.2 pS; Werry et al., 2013; Murray et al., 2016) but having a lower K^+^_o_ dependency compared with KCNQ1 homomers (see below). Our initial whole-cell oocyte recordings of EQQ revealed 26 ± 4% inhibition of current when K^+^_o_ was increased from 5 to 97 mM (Fig. 6 A). Single-channel recordings at identical K^+^_o_ conditions revealed 55 ± 5% reduction in single-channel amplitude at +60 mV (Fig. 6, B–D). Fitting the GHK flux equation to current–voltage data revealed a similar 57 ± 8% reduction in absolute permeability of the single channel (Fig. 6 D). Inhibition of single-channel current by K^+^_o_ was nearly twice as large as on the corresponding whole-oocyte currents. This discrepancy could be attributable to the putative additional effect of external K^+^ on channel gating, flickering, or to the influence of the intracellular factor(s) different in oocytes versus *ltk*-mouse cells.

We next recorded a single F339A mutant channel in order to understand how external K^+^ produces potentiation in this mutant as well as those in V310A and F340A. The average single-channel amplitude of the F339A mutant at 5 mM K^+^_o_ was larger (Fig. 6, E and F) than that of the wild type (Wang et al., 2020), which allowed us to analyze the K^+^_o_ dependency for this mutant. The estimated single-channel amplitude of F339A at +60 mV was nearly unchanged in 97 mM K^+^_o_ conditions compared with that of 5 mM K^+^_o_ (Fig. 6, E and F). Calculations of absolute single-channel permeability via fitting data to the GHK flux equation resulted in values close to those predicted theoretically (Fig. 6 F). These results indicate that alanine substitution at this position eliminates the effect of K^+^_o_ on the unitary conductance of the channel. The current potentiation of F339A mutant by high K^+^_o_ observed in whole-oocyte recordings, therefore, is likely to be attributable to the influence of K^+^_o_ on other channel properties.

### Conductive modes of the KCNQ1 SF and the effect of external K^+^

**Figure 7.**
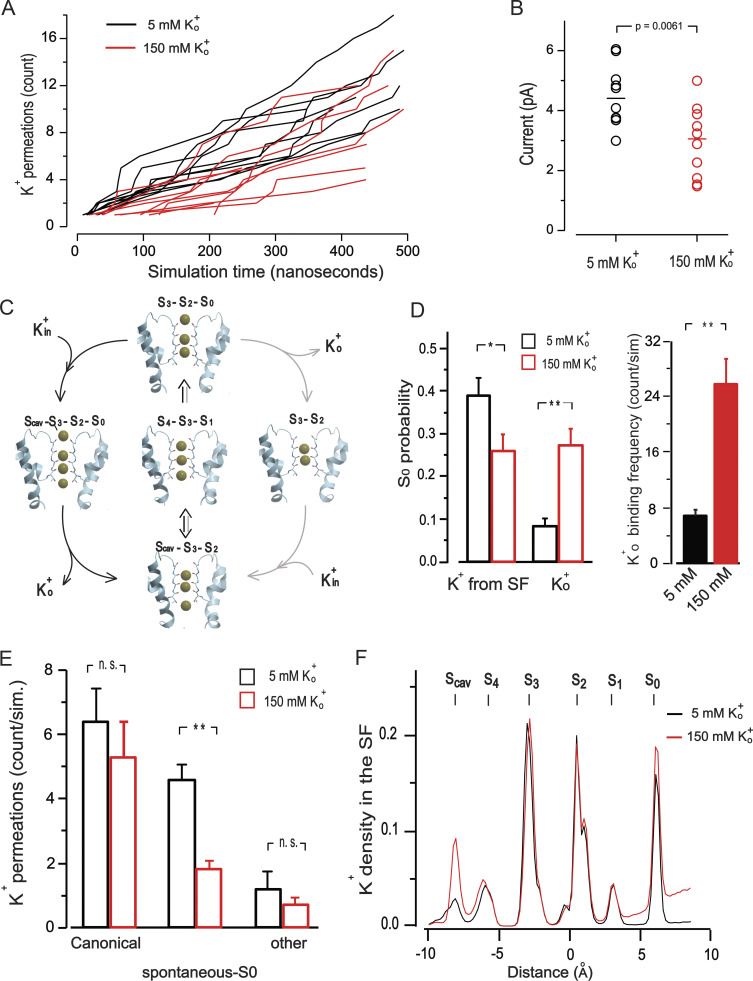
**Modification of SF ion occupancy states by external K**^**+**^**. (A)** Cumulative outward ionic flux at low (5 mM) and high (150 mM) K^+^_o_ conditions. 10 simulations for each K^+^_o_ condition is shown. **(B)** Current amplitudes calculated from the trajectories shown in A. Each point corresponds to the mean current of one simulation. Student’s unpaired *t* test was used to compare the groups. Lines show the median of each group. **(C)** Schematic illustration of canonical (black arrows) and spontaneous-S_0_ release (gray arrows) mechanisms. The key substates of the KCNQ1 SF are shown as snapshots from one representative trajectory at 5 mM K^+^_o_ (trajectory #1). The double-headed arrow denotes the fluctuation between S_cav_-S_3_-S_2_ and S_4_-S_3_-S_1_ substates (see also Fig. S8). **(D)** Occupation probability of the S_0_ site by (1) K^+^ coming from the SF and (2) by external K^+^ (left). Events of K^+^_o_ binding to the S_0_ site of the SF lasting longer than 0.4 nS are counted. **(E)** Distribution of K^+^ permeation events between canonical and spontaneous–S_0_ modes at two different K^+^_o_ conditions. Average of 5 µs (10 simulations) for each concentration is shown. **(F)** Mean K^+^ density in the SF during MD simulations. The center of the mass of G314 Cα atoms is taken as a 0 Z coordinate. S_cav_, K^+^ binding site in the channel cavity. Error bars in the graphs represent ±SEM, *P < 0.05, **P < 0.01, n.s., not significant. Student’s unpaired *t* test was used to compare the groups.

To gain atomic-level insight into how K^+^_o_ affects KCNQ1 current, we next employed a CompEl method (Fig. S6), recently used to explore the mechanism of ion permeation in prototypic K^+^ channels (Köpfer et al., 2014; Kopec et al., 2018). We constructed a similar molecular system with a double-membrane configuration using the atomic coordinates of the pore region together with S4-S5 helix (G245-K354) of the recently determined cryo-EM structure of KCNQ1 (PDB ID: 6V01; Sun and MacKinnon, 2020; see supplementary text at the end of the PDF for details), proposed to represent the open pore conformation. Judged by the RMSD values of the Cα atoms, the stability of the protein in the system (Fig. S7) was comparable with what has been found in early pore-only simulation studies (Bernèche and Roux, 2000; Linder et al., 2013; Bernsteiner et al., 2019). An imbalance of two Cl ions between solute compartments generated a transmembrane potential of ∼300 mV (Fig. S6 and Table S1), which was also in agreement with similar studies on Kv1.2 and KscA channels conducted with the same methodology (Kutzner et al., 2011). Then, MD simulations of 500 ns duration were run at 5 and 150 mM external K^+^ conditions (Fig. S8). Observation of numerous outward K^+^ permeation events at both K^+^_o_ conditions (Fig. 7 A) confirmed that the cryo-EM structure of KCNQ1 with bound PIP2 is the open-conductive conformation of the channel (Sun and MacKinnon, 2020). Calculation of the outward current from the MD trajectories revealed ∼30% reduction of mean current amplitude at 150 mM K^+^_o_ compared with 5 mM conditions (Fig. 7 B). Such inhibition of pore conductance by high K^+^ was qualitatively in agreement with the results obtained in single-channel recordings. Analysis of MD trajectories revealed two principally different K^+^ permeation modes of the SF illustrated schematically in Fig. 7 C and Fig. S8. Nearly half of all K^+^ permeation events at 5 mM K^+^_o_ followed the mechanism described earlier for prototypic K^+^ channels (Jensen et al., 2010; Köpfer et al., 2014; Kopec et al., 2018). According to this mechanism, the potential-driven entering of intracellular K^+^ into the channel internal cavity and its subsequent binding to S_cav_ site triggers a release of the K^+^ bound to the S_0_ site of the SF into the external medium (Fig. 7 C, Fig. S8, and Video 1). By contrast, nearly 40% of the observed K^+^ permeations at 5 mM K^+^_o_ followed a slightly different mechanism that involved a spontaneous release of K^+^ from the S_0_ without any activity at the S_cav_ site (Fig. 7 C, right loop; Fig. S8; and Video 2). We refer to these two K^+^ permeation cycles of the SF as canonical and spontaneous S_0_-release modes, respectively. An important difference between the two modes is the low probability of S_0_ site occupancy by K^+^ in spontaneous S_0_ mode. These permeation modes, along with others that comprise nearly 10% of permeation events in our simulations, randomly interchanged with each other during simulations (Fig. S9). In simulations with high external K^+^, we observed an increase in the frequency and duration of S_0_ site occupancy by K^+^_o_ (Fig. 7 D and Fig. S8), which resulted in a diminution of permeation events occurring via the spontaneous S_0_ mechanism (Fig. 7 E). High K^+^_o_ induced significant redistribution in the SF substates (Fig. S8) with an overall effect of delayed forward relocations of K^+^ ions in the SF. This is reflected in K^+^ density plot as a rise of the S_cav_ peak (Fig. 7 F) and an increase of duration between two subsequent permeations of ions under high external K^+^ conditions (Fig. 7 A, red traces). These occur, for instance, when K^+^_o_ temporarily binds to the vacant S_0_ site during S_4_-S_3_-S_1_ ion occupancy of the SF inducing a backward translocation of K^+^ ions to generate the S_cav_-S_3_-S_2_ substate (Video 3). Further delay in K^+^ outward translocations occurs when K^+^_o_ binds to the S_0_ site of the SF in the S_cav_-S_3_-S_2_ occupancy configuration, resulting in S_cav_-S_3_-S_2_-S_0_ occupancy (Fig. S8 and Video 3). Thus, due to the temporary occupation of the S_0_ site by K^+^_o_, a delay in the outward translocations of K^+^ ion in the SF leads to a reduction of the channel conductance.

**Video 1.** **Movement of K**^**+**^ **ions through the SF via canonical (K**^**+**^**_in_-induced) mechanism.** A fragment from simulations at 5 mM K^+^_o_ conditions. K^+^, Na^+^, and Cl^−^ ions are represented by pink, blue, and cyan spheres, correspondingly. Lipid molecules are shown in gray. Water molecules as well as two channel subunits are omitted for clarity of the view.

**Video 2.** **Permeation of K**^**+**^ **ions through KCNQ1 according to spontaneous S**_**0**_ **mechanism.** A short (43 ns) simulation at 5 mM K^+^_o_ conditions showing the spontaneous release of K^+^ from S_0_ site at S_3_-S_2_-S_0_ occupancy configuration of the SF. Representations of the K^+^, Na^+^, and Cl^−^ ions as well as the lipid molecules are identical to those shown in Video 1.

**Video 3.** **K**^**+**^_**o**_**-induced changes in the SF occupancy substates induced by binding of external K**^**+**^**.** Concentration of the K^+^_o_ is 150 mM. Representation of K^+^, Na^+^, and Cl^−^ ions as well as the lipids are identical to those shown in Video 2.

We next evaluated the dynamic properties of F339, F340, and V310 side chains to understand the K^+^_o_-induced potentiation in corresponding alanine mutants. Remarkably, the aromatic rings of both F339 and F340 adopt two discrete conformations stabilized by distinct interactions with defined residues of the same and the neighboring subunits (Fig. S10). Over the course of the MD simulations, we have observed a flipping of aromatic rings between these two stable configurations in an asymmetric manner (Fig. S10 and Video 4). The aromatic ring of F339 interacts with I268, G269, and T265 residues of the same subunit and L251′ of the neighboring subunit. Upon flipping, the pattern of interaction is changed to comprise L251′, V255′, and L347′ residues of the neighboring subunit (Fig. S10). The benzene ring of F340 is stabilized by V310, G269, and L273 residues of the same subunit (Fig. S10). It makes new hydrophobic and van der Waals contacts with the T311 residue of the SF and I337 as well as V334 residues of the neighboring subunits after flipping. Remarkably, the distribution of probabilities of C-Cα-Cβ-Cγ dihedral angles was changed in high external K^+^ (Fig. S10). These indicate that the F339–F340 region of the protein involved in the stabilization of the lower pore helix is very dynamic in KCNQ1 due to asymmetric flipping of the aromatic rings of different subunits. F339 and F340 residues correspond to the region in prototypic KcsA and *Shaker* K^+^ channels involved in the coupling of the intracellular gate to the SF. Particularly, residues I100–F103 in KcsA and V467–I470 in *Shaker* transmit the conformational changes induced by the wide opening of the gate to the SF leading to C-type inactivation. It has been shown that during this process the side-chain rotameric angle of I100 and F103 residues in KscA undergo significant redistribution (Li et al., 2018). Similar findings have been described for homologous residues (V476 and I470) in *Shaker* (Li et al., 2021). K^+^_o_ sensitivity changes in F339A and F340A mutants observed in our study suggest that a similar interaction may exist in KCNQ1 that shapes the SF properties of the channel. In agreement with this notion, single-channel recordings of F339A have shown an increase in unitary conductance and almost no inhibitory action of K^+^_o_ on this mutant. In addition, the F339A mutant seems to exhibit less flickery phenotype when compared with EQQ (Fig. 6). V310A, F339A, and F340A mutants exhibit slow inactivation phenotype (Fig. 3 A), raising the question of whether the modulatory action of K^+^_o_ could be linked to the slow inactivation process in these mutants. We found that co-expression of KCNE1 eliminates the slow inactivation of F339A (Fig. S11). Remarkably, the potentiating effect of high K^+^_o_ was retained in F339A/KCNE1 heteromeric channels. These results were similar to those obtained previously in R231A/F340W/KCNE1 leak channels (Panaghie et al., 2008). Based on these data, we propose that mutation-induced stabilization of the conductive conformation of the SF is the main cause of the K^+^_o_-sensitivity changes in F339A, F340A, and V310 mutants.

**Video 4.** **The movie depicts the flipping of aromatic rings of F339 and F340 residues.** A snapshot of simulation at low (5 mM) external K^+^ conditions. The side-chains of F339A and F340 are highlighted. The top view from external medium is represented with three K^+^ ions bound to the selectivity filter (pink spheres).

### External K^+^ dependency of heteromeric KCNQ1 channels

**Figure 8.**
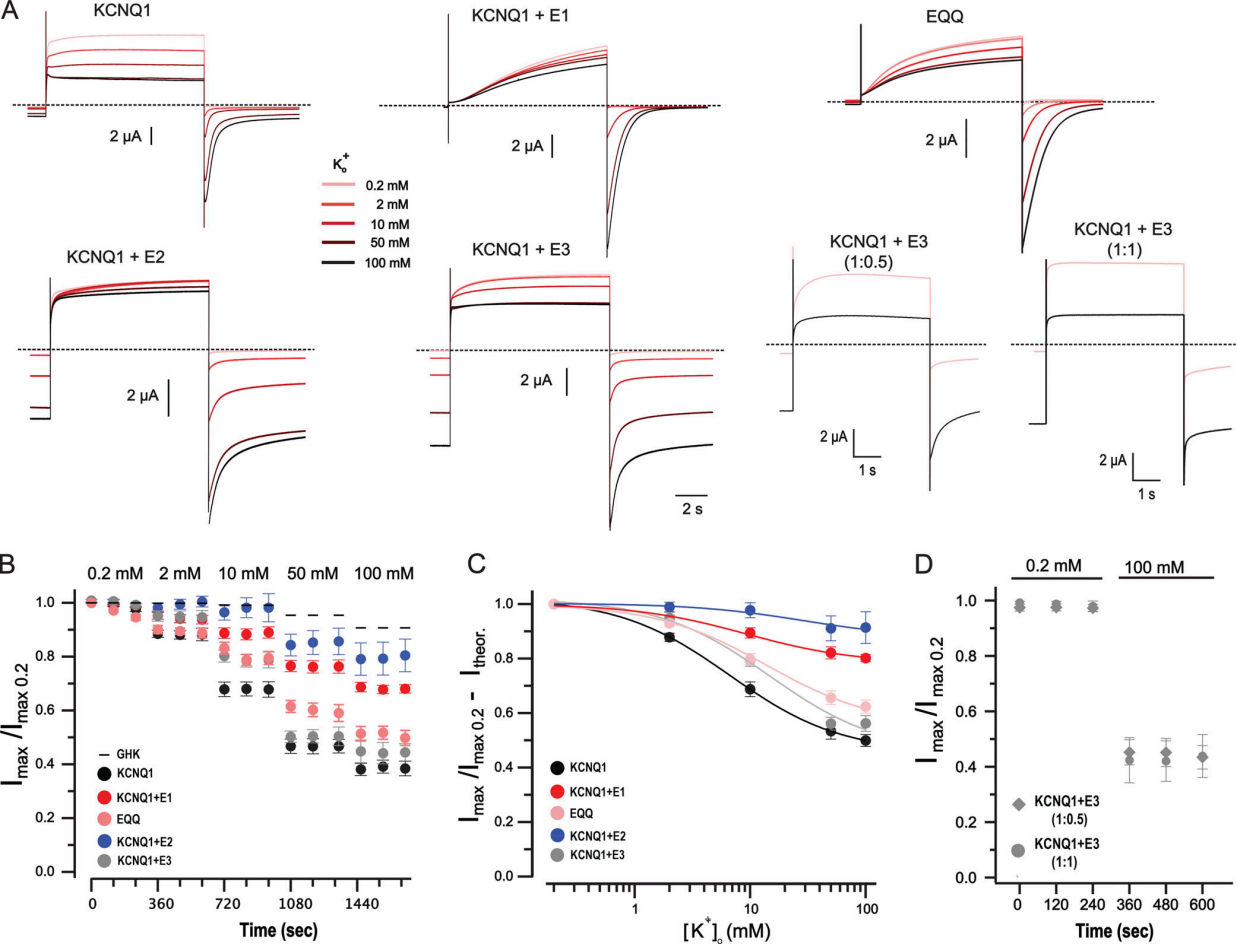
**Influence of [K**^**+**^**]**_**o**_ **on KCNQ1 channels in the absence and presence of KCNE1–3 auxiliary subunits indicated as E1, E2, and E3. (A)** Sample current traces of KCNQ1 channels measured at five different [K^+^]_o_ as indicated. Dashed lines represent 0 current. Oocytes were held at −100 mV and depolarizing pulses of 10 s duration were applied with 120-s interpulse interval. **(B)** Mean maximal current amplitudes at five different concentrations of K^+^_o_ normalized to the first point obtained in 0.2 mM K^+^_o_ (*Imax*/*Imax*_0.2_); *n* = 4–9, error bars represent ±SEM. **(C)** Concentration-dependency of K^+^_o_ inhibition for heteromeric KCNQ1 channels. Data shown in B were corrected for theoretical (Ith) values [(*Imax*/*Imax*_0.2_) − Ith] according to the GHK flux equation, assuming oocyte [K^+^]_in_ of 108 mM (Weber, 1999). Solid lines represent fits of the Hill equation to the data. Mean parameters of the fits are shown in Table 1. **(D)** Inhibition of peak current of the KCNQ1/KCNE3 heteromeric channels injected at 1:0.5 and 1:1 α to β molecular ratio upon elevation of [K^+^]_o_ from 0.2 to 100 mM. Corresponding sample traces are shown in A (lower right); *n* = 4–5, error bars represent ±SEM.

KCNQ1 channel complexes in most human tissues are believed to be heteromeric, composed of pore-forming α subunits and KCNE1–5 β subunits. To evaluate the physiological significance of the above findings, we co-expressed different KCNE proteins with KCNQ1 and analyzed the K^+^_o_ dependency of resultant heteromeric complexes. A marked suppression of K^+^_o_ sensitivity of KCNQ1 was observed upon co-injection of KCNE1 subunit at 1:4 α to β mRNA molecular ratio (Fig. 8 and Table 1) to saturate KCNQ1 with KCNE1. An estimated ∼20% inhibition of current in the range of [K^+^]_o_ from 0.2 to 100 mM is in contrast with the results of Larsen et al. (2011), who reported no effect of K^+^_o_ on KCNQ1/KCNE1 heteromers. One possible reason for this discrepancy is the narrow range of [K^+^]_o_ (1 and 10 mM) used in their study. We then analyzed EQQ tandem channels that correspond to the KCNQ1/KCNE1 heteromers with 4:2 stoichiometry. We revealed marked inhibition by K^+^_o_ in this channel (Fig. 8, A–C). The dose–response curve of EQQ took an intermediate position between those of homomeric and saturated KCNQ1/KCNE1 heteromeric channels (Fig. 8 C and Table 1). Co-expression of KCNE2 with KCNQ1 in oocytes via mRNA injection at 1:4 α to β molecular ratio resulted in voltage-independent channels that were virtually insensitive to K^+^_o_ (Fig. 8, A–C). We observed only 5–9% of inhibition when current amplitudes at 0.2 and 100 mM K^+^_o_ conditions were compared. This was in disagreement with a previously reported ∼30% peak current difference comparing 1 and 10 mM K^+^_o_ conditions in *Xenopus* oocyte expression system (Larsen et al., 2011). A possible reason for such a large discrepancy could be the low levels of KCNE2 co-association with KCNQ1, resulting in nonsaturated KCNQ1/KCNE2 heteromers in this latter study as the small voltage-independent current fraction and fast deactivation kinetics of tail currents indicate. For the KCNQ1/KCNE3 complex, we observed a maximal inhibition (*I*_max_) comparable with that of the homomeric KCNQ1 channel and a statistically significant right-shift in the dose–response curve (Fig. 8 and Table 1). A small fractional voltage-dependent activation component visible in traces corresponding to 0.2 and 2 mM K^+^_o_ pointed toward the possibility that KCNQ1 is not fully saturated by KCNE3 subunit despite the injection of mRNA at 1:1 α to β molecular ratio. Further fractional increase of mRNA encoding KCNE3 causes a significant deterioration of oocytes. Furthermore, the assessment of the dose response in the KCNQ1/KCNE3 leak channel was a difficult experimental goal in our study even at 1:1 molecular ratio since the long-lasting recordings of these channels became unstable during the time needed due to large inward holding currents. Therefore, in another set of experiments, we varied the α to β ratio and compared the current amplitudes at 0.2 and 100 mM K^+^_o_ concentrations (Fig. 8, A and D). These experiments have clearly shown that an increase in KCNE3 ratio markedly changed the gating kinetics of the channel, but the inhibitory action of K^+^_o_ was comparable. The extent of inhibition observed in these experiments with injected 1:1 molecular ratio is comparable with data derived from the dose–response analysis (Fig. 8 B), confirming the significant K^+^_o_ sensitivity of KCNQ1/KCNE3 heteromers. Given that both KCNQ1/KCNE2 and KCNQ1/KCNE3 mediate similar potential-independent currents in the physiological range of voltages (Schroeder et al., 2000; Tinel et al., 2000), the observed difference in K^+^_o_ sensitivity between these channels is remarkable. Overall, the results indicate that, along with homomeric KCNQ1, various KCNQ1/KCNE complexes are regulated by external K^+^. Our results and those of earlier studies (Larsen et al., 2011; Wang et al., 2015) show that the extent of the K^+^_o_-dependent regulation of KCNQ1/KCNE complexes may depend on the type and the quantity of the associated β subunits.

## Discussion

K^+^_o_ exerts a stabilizing influence on the conductive conformation (Doi et al., 1996; Cohen et al., 2008; Sandoz et al., 2009; Massaeli et al., 2010; Edvinsson et al., 2011) in most K^+^ channels, a typical example of which is the attenuation of C-type inactivation in a large number of K^+^ channels (Pardo et al., 1992; López-Barneo et al., 1993; Kiss and Korn, 1998; Zhou et al., 2001; Cuello et al., 2010). K^+^_o_ is obligatory for the conductive SF conformation of HERG channels and their membrane availability (Massaeli et al., 2010). Although the mechanism and structural particularities of C-type inactivation of K^+^ channels are not completely understood (Liu et al., 2015; Armstrong and Hollingworth, 2018), the bulk of data suggests that it involves conformational changes in the external mouth of the channel pore (Kurata and Fedida, 2006; Armstrong and Hoshi, 2014; Pau et al., 2017; Tan et al., 2022). Importantly, at the single channel level, C-type inactivation is the decline of the channel’s open probability over time (Yang et al., 1997; Cuello et al., 2010; Pau et al., 2017), implying that external K^+^ shifts the open–closed equilibrium of the pore toward open states without any influence on unitary conductance. In notable contrast, here, we report a marked reduction of unitary conductance of KCNQ1 under high external K^+^ conditions.

Alanine mutation in three residues G348, F350, and K354 that are located in the gate region markedly reduced the inhibitory effect of K^+^_o_ (Fig. 3 C), pointing toward the possible involvement of the cytoplasmic gate in the inhibition process. Following lines of evidence, yet, suggest that the K^+^ sensitivity of KCNQ1 is largely independent of the conformational state of the gate: (1) constitutively open KCNQ1/KCNE3 heteromeric channels displays K^+^_o_ sensitivity comparable to wild type homomers (Fig. 8); (2) ∼30% inhibition of current amplitude observed in MD simulations with the intracellular gate “clamped” at open conformation (Fig. 7 B) is comparable with that of homomeric KCNQ1 (∼28%) when 5 and 100 mM concentrations are compared (Fig. 1 E); (3) G348A mutation induces a shift in conductance–voltage relationship toward hyperpolarized potentials (ΔV1/2 _G348A_ ∼ −10 mV), whereas F350A and K354A exhibit positive GV shifts (ΔV1/2 _F350A_ ∼ +20 mV, ΔV1/2 _K354A_ ∼ +15 mV; Ma et al., 2011). The K^+^_o_ sensitivity of all these mutants is changed in the same direction (Fig. 3 C). Conversely, marked gating perturbations observed in alanine mutants of S349 and L353 residues (ΔV1/2 _S349A_ ∼ +20 mV, ΔV1/2 _K353A_ ∼ −20 mV; Ma et al., 2011) do not match with unchanged K^+^_o_ sensitivity of these channels (Fig. 3 C). The side chains of S349 and L353 form the cytoplasmatic seal according to available cryo-EM structures (Sun and MacKinnon, 2017, 2020), confirming the notion that no correlation between steady-state gating of the channel and its K^+^_o_ dependency exists. How could G348A, F350A, and K354A mutations change the K^+^_o_ sensitivity of the channel? The cytoplasmatic gate of many K^+^ channels is allosterically coupled to the SF (Ader et al., 2009; Li et al., 2018; Kopec et al., 2019; Li et al., 2021). Amino acids I100–F103 in KcsA and V467–I470 in *Shaker* prototypic channels play an important role in such coupling (Li et al., 2018; Li et al., 2021). The analogous I337–F340 region in KCNQ1 having a high mutational impact on K^+^_o_ sensitivity may be involved in a similar coupling process that transmits conformational changes due to G348A, F350A, and K354A mutations to the SF influencing its function.

K^+^_o_ differentially affects the function of the SF in KCNQ1 versus KcsA channels causing a reduction of conductance in one case and a slowing of the inactivation in the other. One possible explanation could be the asymmetricity of interactions in I337–F340 region of the KCNQ1 channel. I100–F103–T74 interactions in KcsA that play an essential role in the gate–SF coupling of the channel are symmetrical. Analogous interactions of homologous residues I337–T311–F340 in KCNQ1 are asymmetric. They take place in one (S1) subunit at a much higher probability (Fig. S10) at the dihedral angle of F340 side chain χ = 20–80^o^. In the other three subunits, the G269–V310–F340 interactions prevail at dihedral angle χ = 120–180^o^. Furthermore, F339 residue in KCNQ1 is substituted with serine 102 in KcsA and threonine 469 in *Shaker*. The large side-chain volume at this position with highly dynamic properties (Fig. S10) might be another contributing factor. Overall, these data indicate that the allosteric coupling of the KCNQ1 inner gate to the selectivity could be highly dynamic in KCNQ1. Certain mutations in the gate region such as G348A, F350A, and K354A may strengthen coupling, leading to changes in the SF properties.

The results of MD simulations suggest that the flipping of F339 and F340 aromatic rings most likely destabilizes the conductive dynamics of the KCNQ1 SF, which may be further deteriorated by the binding and unbinding of external K^+^ at high K^+^_o_ conditions. Single-channel analysis shows that the removal of the aromatic ring by F339A mutation increased the unitary conductance and almost completely eliminated the effect of K^+^_o_ on unitary conductance (Fig. 6). A large mutational effect observed by alanine substitution of V310 is likely to be due to modification of the V310–F340 interactions (Fig. 3 D and Fig. S10).

In our study, current amplitudes calculated from MD trajectories are about 8-fold higher than the average single-channel amplitudes of EQQ and, at least, 20-fold higher than corresponding single-channel currents of KCNQ1 homomers. The factors contributing to this discrepancy may include, but are not limited to, applied supraphysiological voltages, imperfections in force field parameters, truncation of the channel to pore-only structures, and movement restrictions applied to keep the channels in the open state. Many aspects of these limitations of MD methods are covered in recent reviews (Flood et al., 2019; Mironenko et al., 2021). Despite these limitations, MD simulations translate the modulatory effect of the external K^+^ on unitary conductance reasonably well and provided atomic insights into the mechanism underlying this phenomenon.

Co-expression of KCNE1–3 subunits with KCNQ1 at 1:1 and higher β to α mRNA ratios almost completely eliminated the fast inactivation of KCNQ1. Nevertheless, we observed a differential influence of these β subunits on the K^+^_o_ dependency of KCNQ1. This confirmed our findings of alanine screening experiments (Fig. 2) on the heteromeric channel level. How does the co-expression of KCNE subunits change the K^+^_o_ sensitivity of KCNQ1? Early studies have shown that the extracellular region of KCNE1 interacts with external residues of S6 segments of the KCNQ1 pore (Xu et al., 2008; Chung et al., 2009). Whether these interactions contribute to the increased single-channel conductance in KCNQ1/KCNE1 heteromers (Sesti and Goldstein, 1998; Murray et al., 2016) remains unknown. The involvement of the SF in this process is also unclear. We consider it likely that KCNE1 stabilizes the conductive dynamics of the SF leading to an increase in unitary conductance and the reduction of the K^+^_o_ sensitivity of the channel. KCNE2 accessory subunits affect both the gating (Tinel et al., 2000) and the plasma membrane targeting of KCNQ1 (Roura-Ferrer et al., 2010). Almost complete elimination of the K^+^_o_ sensitivity of KCNQ1 by KCNE2 coexpression confirms the action of KCNE2 on the SF proposed previously based on the measurements of Rb^+^/K^+^ permeability ratio in KCNQ1/KCNE2 heteromers (Wang et al., 2012). Co-assembly of KCNE3 with KCNQ1 shifts the dose–response curve of the channel toward higher K^+^_o_ concentrations (Fig. 8) indicating either a decrease of K^+^_o_ affinity to S_0_ or a lower efficacy of the inhibition following the K^+^_o_ binding to S_0_. A recent KCNQ1/KCNE3 structure (Sun and MacKinnon, 2020) together with earlier crosslinking electrophysiological investigations suggests that the pattern of interaction of the extracellular region of KCNE3 with KCNQ1 differs from that of KCNE1. The differential effect of K^+^_o_ on heteromeric KCNQ1/KCNE3 compared with that of KCNQ1/KCNE1 channels, therefore, could be attributable to distinct conformational differences in the upper pore region of these two channels. Comparison of available atomic resolution structures of KCNQ1 and KCNQ1/KCNE3 channels reveals no discernable structural difference(s) in the SF (Sun and MacKinnon, 2020), suggesting that the shift of the dose–response curve is likely attributable rather to the dynamic properties of the SF.

### Physiological significance

In mammals, KCNQ1 and KCNE1 proteins are expressed in the apical surfaces of MCs of the stria vascularis (Knipper et al., 2006) and the dark cells of vestibular organs (Nicolas et al., 2001). In both cell types, the extracellular side of the channels is exposed to endolymph, the K^+^ concentration of which changes from the low millimolar range during embryonic development (Anniko and Wersäll, 1979) to 150 mM in adults (Hibino and Kurachi, 2006). The extent of reported K^+^_o_-induced I_ks_ current reduction in mouse MCs (Wang et al., 2015) is comparable with what we have observed for homomeric KCNQ1 and heteromeric EQQ channels (Fig. 8). This suggests a KCNQ1:KCNE1 stoichiometry lower or equal to 4:2 in these MCs. An elegant study by Kurachi and coworkers has shown that the K^+^ diffusion through the apical membrane of MCs mediated by I_ks_ channels contributes to the endocochlear potential (EP; Nin et al., 2008). The voltage-dependent regulation of I_ks_ in these cells will be partially or completely lost since these channels experience membrane potentials close to +10 mV (Offner et al., 1987) or much higher (+80 mV) according to a more recent study (Nin et al., 2008). K^+^_o_ sensitivity of KCNQ1/KCNE1 under these conditions will be a powerful mechanism for regulation of K^+^ flow toward endolymph serving as an important feedback regulatory tool for maintenance of the high endolymph [K^+^]. Such regulation can be particularly pronounced during the period from prehearing to hearing onset when [K^+^] in the endolymph gradually increases. Since K^+^ diffusion through the apical membrane of MCs contributes to the generation of the endocochlear potential (Nin et al., 2008), the K^+^_o_-sensitivity of KCNQ1 is essential for hearing and balance.

The stoichiometric ratio for KCNQ1/KCNE in human cardiomyocytes is currently uncertain. It is becoming increasingly clear that it can vary between 4:1 and 4:4 depending on the availability of KCNE1 subunits as evidenced in heterologous expression studies (Nakajo et al., 2010; Murray et al., 2016). Our estimation of K^+^_o_-induced I_ks_ reduction for various heteromers under the severe hypokalemic condition with reported serum [K^+^] as low as 1.2 mM (Kratz et al., 2004; Garcia et al., 2008) is <15% compared with normal serum K^+^ (3.5–5.5 mM). At first glance, such a small modulation of I_ks_ implies no large effect on cardiac excitability. Nevertheless, one must consider that hypokalemia significantly reduces I_kr_ current in cardiomyocytes mediated by HERG channels (Massaeli et al., 2010), suggesting that even a minor reduction of I_ks_ under these circumstances may have a significant impact. At present, the cellular expression, localization, and subunit composition of KCNQ1 channels in other organs, such as in stomach, kidney, pancreas, thyroid, and testis are far from known, leaving the full physiological significance of the described phenomenon as an open question for future studies.

## Supplementary Material

Table S1 shows the summary of the computational electrophysiology simulations.

Table S2 shows the P values of statistically significant findings.
