# Supplementary material for: Mechanism of external K+ sensitivity of KCNQ1 channels
Source: J Gen Physiol. 2023 Feb 21;155(5):e202213205. doi: 10.1085/jgp.202213205 (PMC9960071; doi:10.1085/jgp.202213205)
Supplement: Table S1 — shows the summary of the computational electrophysiology simulations. [file JGP_202213205_TableS1.docx]

**Table S1. The summary of the computational electrophysiology simulations.**

| **Channel** | **KCNQ1** | |
| --- | --- | --- |
|  | 5 mM K^+^_o_ | 150 mM K^+^_o_ |
| Voltage | ~320 | ~290 |
| Ions inside | 27 K^+^ | 27 K^+^ |
| Ions outside | 2 K^+^ /23 Na^+^ | 27 K^+^ |
| Initial ions in the selectivity filter | 3K^+^ | 3K^+^ |
| lipids | 216 | |
| Force field | Amber ff14SB | |
| Ions | Joung and Cheatham for TIP3P | |
| Lipids | Amber lipid17 POPC | |
| **Simulations** | | |
| Independent simulations | 10 | 10 |
| Total simulation time (ns) | 5000 | 5000 |
