# Supplementary material for: Mechanism of external K+ sensitivity of KCNQ1 channels
Source: J Gen Physiol. 2023 Feb 21;155(5):e202213205. doi: 10.1085/jgp.202213205 (PMC9960071; doi:10.1085/jgp.202213205)
Supplement: Table S2 — shows the P values of statistically significant findings. [file JGP_202213205_TableS2.docx]

**Table S2. P-values of statistically significant findings.**

| **Item** | | **Comparison** | **P-values** | | |
| --- | --- | --- | --- | --- | --- |
| Figure 2 | B | WT vs E290A/S292AE395A | *** | 0.0003 | ANCOVA |
|  | E | WT vs E290A (*I*_max_) | ** | 0.0062 | 1 way ANOVA Dunnett‘s |
|  |  | WT vs S291A (*I*_max_) | * | 0.0383 | 1 way ANOVA Dunnett‘s |
|  |  | WT vs E295A (*I*_max_) | ** | 0.0049 | 1 way ANOVA Dunnett‘s |
|  | F | WT vs E290R (*I*_max_) | ** | 0.0011 | 1 way ANOVA Dunnett‘s |
|  |  | WT vs E290Q (*I*_max_) | ** | 0.0032 | 1 way ANOVA Dunnett‘s |
|  |  | WT vs E290Q (IC_50_) | * | 0.0279 | 1 way ANOVA Dunnett‘s |
| Figure 3 | C | WT vs E261A | * | 0.0210 | 1 way ANOVA Dunnett‘s |
|  |  | WT vs I268A | *** | 0.0001 | 1 way ANOVA Dunnett‘s |
|  |  | WT vs G269A | *** | 0.0001 | 1 way ANOVA Dunnett‘s |
|  |  | WT vs I274A | * | 0.0148 | 1 way ANOVA Dunnett‘s |
|  |  | WT vs E290A | *** | 0.0001 | 1 way ANOVA Dunnett‘s |
|  |  | WT vs S291A | * | 0.0145 | 1 way ANOVA Dunnett‘s |
|  |  | WT vs E295A | * | 0.0334 | 1 way ANOVA Dunnett‘s |
|  |  | WT vs V310A | *** | 0.0001 | 1 way ANOVA Dunnett‘s |
|  |  | WT vs V324A | *** | 0.0001 | 1 way ANOVA Dunnett‘s |
|  |  | WT vs F335A | ** | 0.0072 | 1 way ANOVA Dunnett‘s |
|  |  | WT vs I337A  WT vs S338A | ***  * | 0.0001  0.0352 | 1 way ANOVA Dunnett‘s  1 way ANOVA Dunnett‘s |
|  |  | WT vs F339A | *** | 0.0001 | 1 way ANOVA Dunnett‘s |
|  |  | WT vs F340A | *** | 0.0001 | 1 way ANOVA Dunnett‘s |
|  |  | WT vs L342A | *** | 0.0001 | 1 way ANOVA Dunnett‘s |
|  |  | WT vs G348A | *** | 0.0001 | 1 way ANOVA Dunnett‘s |
|  |  | WT vs G350A | *** | 0.0001 | 1 way ANOVA Dunnett‘s |
|  |  | WT vs K354A | *** | 0.0001 | 1 way ANOVA Dunnett‘s |
| Figure 7 | D | 5 mM K^+^ vs 150 mM K^+^ (K^+^ from SF) | * | 0.0271 | Unpaired Students t-test |
|  |  | 5 mM K^+^ vs 150 mM K^+^ ( K^+^_o_) | ** | 0.0011 | Unpaired Students t-test |
|  |  | 5 mM K^+^ vs 150 mM K^+^ (K^+^_o_ frequency) | ** | 0.0042 | Unpaired Students t-test |
|  | E | 5 mM K^+^ vs 150 mM K^+^  (spontaneous S0) | *** | 0.0001 | Unpaired Students t-test |
| Table 1 | | WT *I*_max_ vs Q1+E1 *I*_max_ | *** | 0.0003 | 1 way ANOVA Dunnett‘s |
|  |  | WT *I*_max_ vs EQQ *I*_max_ | ** | 0.0049 | 1 way ANOVA Dunnett‘s |
|  |  | WT *I*_max_ vs Q1+E2 *I*_max_ | *** | 0.0007 | 1 way ANOVA Dunnett‘s |
|  |  | WT IC_50_ vs Q1+E3 IC_50_ | * | 0.0251 | 1 way ANOVA Dunnett‘s |
|  |  | WT *I*_max_ vs E261A *I*_max_  WT IC_50_ vs E261A IC_50_ | *  ** | 0.0417  0.0092 | 1 way ANOVA Dunnett‘s  1 way ANOVA Dunnett‘s |
|  |  | WT *I*_max_ vs I274A*I*_max_ | ** | 0.0082 | 1 way ANOVA Dunnett‘s |
|  |  | WT *I*_max_ vs E290A *I*_max_ | ** | 0.0076 | 1 way ANOVA Dunnett‘s |
|  |  | WT *I*_max_ vs E290R *I*_max_ | ** | 0.0044 | 1 way ANOVA Dunnett‘s |
|  |  | WT IC_50_ vs E290Q IC_50_ | ** | 0.0083 | 1 way ANOVA Dunnett‘s |
|  |  | WT *I*_max_ vs E290Q *I*_max_ | ** | 0.0098 | 1 way ANOVA Dunnett‘s |
|  |  | WT *I*_max_ vs S291A *I*_max_ | ** | 0.0279 | 1 way ANOVA Dunnett‘s |
|  |  | WT *I*_max_ vs S295A *I*_max_ | ** | 0.0014 | 1 way ANOVA Dunnett‘s |
|  |  | WT IC_50_ vs F335A IC_50_ | * | 0.0193 | 1 way ANOVA Dunnett‘s |
|  |  | WT *I*_max_ vs F335A *I*_max_ | *** | 0.0001 | 1 way ANOVA Dunnett‘s |
|  |  | F351A K^+^ vs F351A Rb^+^ (IC_50_) | *** | 0.0004 | 1 way ANOVA Dunnett‘s |
|  |  | F351A K^+^ vs F351A Rb^+^ (*I*_max_) | *** | 0.0001 | 1 way ANOVA Dunnett‘s |
